# Toward a unifying framework for the modeling and identification of motor primitives

**DOI:** 10.3389/fncom.2022.926345

**Published:** 2022-09-12

**Authors:** Enrico Chiovetto, Alessandro Salatiello, Andrea d'Avella, Martin A. Giese

**Affiliations:** ^1^Section for Computational Sensomotorics, Centre for Integrative Neuroscience, Hertie Institute for Clinical Brain Research, University Clinic Tübingen, Tübingen, Germany; ^2^Laboratory of Neuromotor Physiology, IRCCS Fondazione Santa Lucia, Rome, Italy; ^3^Department of Biomedical and Dental Sciences and Morphofunctional Imaging, University of Messina, Messina, Italy

**Keywords:** motor primitives, muscle synergies, Fourier-based Anechoic Demixing Algorithm (FADA), anechoic mixture model, dimensionality reduction, motor redundancy

## Abstract

A large body of evidence suggests that human and animal movements, despite their apparent complexity and flexibility, are remarkably structured. Quantitative analyses of various classes of motor behaviors consistently identify spatial and temporal features that are invariant across movements. Such invariant features have been observed at different levels of organization in the motor system, including the electromyographic, kinematic, and kinetic levels, and are thought to reflect fixed modules—named motor primitives—that the brain uses to simplify the construction of movement. However, motor primitives across space, time, and organization levels are often described with *ad-hoc* mathematical models that tend to be domain-specific. This, in turn, generates the need to use model-specific algorithms for the identification of both the motor primitives and additional model parameters. The lack of a comprehensive framework complicates the comparison and interpretation of the results obtained across different domains and studies. In this work, we take the first steps toward addressing these issues, by introducing a unifying framework for the modeling and identification of qualitatively different classes of motor primitives. Specifically, we show that a single model, the anechoic mixture model, subsumes many popular classes of motor primitive models. Moreover, we exploit the flexibility of the anechoic mixture model to develop a new class of identification algorithms based on the Fourier-based Anechoic Demixing Algorithm (FADA). We validate our framework by identifying eight qualitatively different classes of motor primitives from both simulated and experimental data. We show that, compared to established model-specific algorithms for the identification of motor primitives, our flexible framework reaches overall comparable and sometimes superior reconstruction performance. The identification framework is publicly released as a MATLAB toolbox (FADA-T, https://tinyurl.com/compsens) to facilitate the identification and comparison of different motor primitive models.

## 1. Introduction

The motor system controls a large number of degrees of freedom of the musculoskeletal system through a hierarchical architecture (Bernstein, 1947; d'Avella et al., 2015; Merel et al., 2019). It has been proposed that at the lower levels of the hierarchy, fixed modules—often referred to as motor primitives—reduce the complexity of the control problem, simplifying both motor control and learning (Tresch et al., 1999; Flash and Hochner, 2005; Bizzi et al., 2008; Bizzi and Ajemian, 2020; Cheung and Seki, 2021). Convincing evidence for the existence of motor primitives is provided by the consistent observation of temporal and spatial regularities at different levels of the control hierarchy. For example, such regularities have been reported at the level of the motor cortex (e.g., Overduin et al., 2015; Kadmon Harpaz et al., 2019), spinal interneurons (e.g., Hart and Giszter, 2010; Levine et al., 2014; Takei et al., 2017), motor neurons (e.g., d'Avella et al., 2003; Ivanenko et al., 2004; Torres-Oviedo et al., 2006), joint kinetics (e.g., Mussa-Ivaldi and Giszter, 1992; Santello and Soechting, 2000; Thomas et al., 2005), and joint kinematics (e.g., Santello et al., 1998; Kaminski, 2007; Chiovetto and Giese, 2013).

However, the heterogeneity of domains and observation levels has led to the development of a variety of computational models to explain how the observed regularities are plausibly generated by the underlying fixed modules. Such variety, in turn, has created the need to use different identification algorithms that sometimes have to be devised *ex novo*. For example, motor primitives at the electromyographic (EMG) level are typically extracted with non-Negative Matrix Factorization (NMF—e.g., Tresch et al., 1999; Ting and Macpherson, 2005; Godlove et al., 2016), while motor primitives at the kinematic level are often extracted with Principal Component Analysis (PCA—e.g., Santello et al., 1998; Kaminski, 2007; Chiovetto et al., 2012), Independent Component Analysis (ICA—e.g., Mori and Hoshino, 2002; Lambert-Shirzad and Van der Loos, 2017), or Factor Analysis (FA—e.g., Smith et al., 2006; Steinberg and Bock, 2013). Alternative approaches are also common: for example, ICA has also been successfully used to extract motor primitives at the EMG level (e.g., Hart and Giszter, 2004; Ivanenko et al., 2005; Dominici et al., 2011). More complex models, which introduce trial-dependent delays in the motor primitives activation (Omlor and Giese, 2006) and try to simultaneously capture both temporal and spatial primitives (d'Avella et al., 2003; Delis et al., 2014), require specialized identification algorithms. This multitude of mathematical models and identification algorithms complicates the comparison of the results from different studies: in the absence of a standardized framework for the definition and identification of motor primitives, it is hard to assess whether potential differences observed between studies are due to the use of a different model, identification algorithm, or genuine experimental manipulations.

To simplify such comparative analysis, we introduce here a new unifying framework to model and identify several popular classes of motor primitive models. Specifically, we first show that common models of spatial, temporal, and spatiotemporal modularity, with and without delays, can be considered as special cases of a single generative model: the *anechoic mixture model*. We then introduce a new class of identification algorithms, which we derived extending the *Fourier-based Anechoic Demixing Algorithm* (FADA—Chiovetto and Giese, 2013) to fit all the considered modularity models. Finally, we validate our framework by showing that it can robustly extract different classes of motor primitives from both simulated and experimental data with an accuracy that is comparable and sometimes superior to that achieved using model-specific identification methods.

## 2. Methods

### 2.1. Generative models of spatial and temporal regularities

This section provides a brief survey of the most common models of the modular organization of motor behavior. In general, such models explain the spatial and temporal invariances observed during movements as arising from spatial and temporal modules that are fixed across trials. Such modules are typically referred to as motor primitives or synergies. In the following, we will assume that the activity patterns of *M* degrees of freedom (DOFs) recorded during the execution of one of *L* different trials, over *T* time samples, are collected in an *M* by *T* matrix **X**^*l*^, where *l* is the trial index. Depending on the model, it will sometimes be useful to refer to individual column vectors **x**^*l*^(*t*), or to individual entries $x_{m}^{l}\left(t\right)$ of this matrix. Depending on the context, the DOFs represent different electromyographic (EMG), kinetic, or kinematic signals. Signals relative to different trials are considered to have a fixed duration *T*_*s*_ and to be sampled with a constant sampling frequency.

#### 2.1.1. The spatial decomposition model

One classical definition of motor primitives is based on the observation, during rhythmic and goal-directed movements, of specific covariation patterns between different DOFs that are invariant across time and trials. Such fixed covariation patterns are typically interpreted as reflecting the coordinated recruitment of multiple muscles or joints. This type of model has been particularly successful at explaining regularities in EMG signals (Tresch et al., 1999; Ting and Macpherson, 2005; Torres-Oviedo et al., 2006). Consistent with these observations is the following generative model:


(1) $$\begin{array}{r} {\text{x}}^{l}\left(t\right)=\overset{P}{\underset{p=1}{\sum }}{\text{w}}_{p}\cdot c_{p}^{l}\left(t\right)+residuals \end{array}$$

In this equation, the vectors **x**^*l*^(*t*) collect the values taken on by all the DOFs at time point *t* (assuming discrete time steps, 1 ≤ *t* ≤ *T*) in trial number *l*. The column vectors **w**_*p*_ capture the invariant *spatial patterns* and thus represent the motor primitives themselves. The number of primitives is *P*, and the scalars $c_{p}^{l}\left(t\right)$ are the time-dependent mixing weights of the primitives. Mixing weights (and residuals) can generally vary across trials. Importantly, the components of this model are often assumed to be non-negative [i.e., $c_{p}^{l}\left(t\right)\geq 0$ and *w*_*p,m*_ ≥ 0]. This assumption is particularly common when the model is used to explain EMG data, which typically consist of time series of non-negative signals [i.e., $x_{m}^{l}\left(t\right)\geq 0$, ∀*m, l*] reflecting the excitatory activity of underlying motoneurons (Farina et al., 2014). As this model is based on the invariant patterns in the spatial domain (i.e., in the DOF space), it is often referred to as spatial decomposition model.

#### 2.1.2. The temporal decomposition model

An alternative definition of motor primitives is based on the observation of invariant covariation patterns across time, which are thought to represent the activity of latent temporal source functions *s*_*p*_(*t*). Temporal components based on this definition have been identified in kinematic (Kaminski, 2007; Berret et al., 2009; Chiovetto and Giese, 2013), kinetic (Thomas et al., 2005), and EMG (Ivanenko et al., 2004, 2005; Chiovetto et al., 2010) space. The underlying generative model, which we will refer to as temporal decomposition model is defined by:


(2) $$\begin{array}{l} x_{m}^{l}\left(t\right)=\overset{P}{\underset{p=1}{\sum }}c_{mp}^{l}\cdot s_{p}\left(t\right)+residuals \end{array}$$

In this equation, $x_{m}^{l}\left(t\right)$ is the value of the *m*-th DOF at time *t* in trial number *l*, and the corresponding scalar mixing weights $c_{mp}^{l}$ change between different trials. The *P* temporal primitives *s*_*p*_(*t*), however, are assumed to be invariant over trials. Both the spatial (1) and the temporal (2) decomposition models assume that the latent sources affect the activity patterns of all the different degrees of freedom simultaneously, that is, without any DOF-specific delays. For this reason, such models are sometimes referred to as synchronous decomposition models.

#### 2.1.3. The temporal decomposition model with delays

An alternative to the synchronous decomposition models to explain temporal regularities has been proposed by Omlor and Giese (2006, 2007, 2011). This model allows for delayed activation of the temporal basis functions, where the delays can potentially vary across different primitives, DOF, and trials. This can be interpreted as reflecting, for example, delays between the activation of different muscles within the same temporal primitive. Mathematically, this model is characterized by the equations:


(3) $$\begin{array}{l} x_{m}^{l}\left(t\right)=\overset{P}{\underset{p=1}{\sum }}c_{mp}^{l}\cdot s_{p}\left(t-\tau_{mp}^{l}\right)+residuals \end{array}$$

Importantly, in this model, the time delays $\tau_{mp}^{l}$ and mixing weights $c_{mp}^{l}$ can vary over trials, while the basis functions *s*_*p*_(*t*) are invariant, as in model (2). Like for model (1), inequality constraints can be imposed on the mixing weights and source functions of models (2) and (3) to account for the non-negativity of EMG signals.

#### 2.1.4. The spatiotemporal decomposition model

The models discussed so far, defined by the Equations (1)–(3) can only account for regularities in the spatial or temporal domain, but not both. To deal with such a limitation, d'Avella and Tresch (2002), d'Avella et al. (2003, 2006) introduced the concept of spatiotemporal (or time-varying) primitives, which can be considered as latent spatiotemporal activity patterns that are invariant over trials. The resulting spatiotemporal decomposition model thus assumes that the activity patterns measured on the DOFs are generated by mixing such primitives with trial-specific weights. Such latent sources, or primitives, can also be shifted in time by a trial-specific delay. This results in the following generative model:


(4) $$\begin{array}{l} {\text{x}}^{l}\left(t\right)=\overset{P}{\underset{p=1}{\sum }}c_{p}^{l}\cdot {\text{w}}_{p}\left(t-\tau_{p}^{l}\right)+residuals \end{array}$$

Note that in this model, unlike in model (3), mixing weights $c_{p}^{l}$ and delays $\tau_{p}^{l}$ do not change across muscles. The vector-valued source functions **w**_*p*_(*t*) are invariant across trials and represent the spatiotemporal primitives. Such primitives and the corresponding mixing weights have typically been assumed to be non-negative (d'Avella et al., 2003), although also models with unconstrained parameters have been applied to model phasic EMG activity (d'Avella et al., 2006).

#### 2.1.5. The space-by-time decomposition model

An alternative approach to simultaneously model spatial and temporal regularities was introduced by Delis et al. (2014). This model, named space-by-time decomposition model, assumes the simultaneous existence of *P*_*sp*_ spatial and *P*_*tp*_ temporal primitives that, unlike in model (4), are not grouped in *P* spatio-temporal primitives, but are free to vary independently.


(5) $$\begin{array}{l} {\text{x}}^{l}\left(t\right)=\overset{P_{tp}}{\underset{p=1}{\sum }}\overset{P_{sp}}{\underset{q=1}{\sum }}s_{p}\left(t-\tau_{pq}^{l}\right)\cdot c_{pq}^{l}\cdot {\text{w}}_{q}+residuals \end{array}$$

In this model, **w**_*q*_ and *s*_*p*_(*t*) define the trial-independent spatial and temporal primitives as in models (1) and (2), while the mixing weights $c_{pq}^{l}$ and time delays $\tau_{pq}^{l}$ are trial-dependent. Since the model was originally designed to account for EMG data, all parameters are typically assumed to be non-negative, with the exception of the time delays.

### 2.2. The unifying anechoic mixture model

All previously discussed models can be derived as special cases of a single model: the *anechoic mixture model*. This type of model is popular in acoustics, where it is applied for modeling acoustic mixtures in reverberation-free rooms (Torkkola, 1996; Emile and Comon, 1998; Bofill, 2003; Yilmaz and Rickard, 2004). This model assumes typically a set of *R* recorded acoustic signals *y*_*r*_(*t*) that are created by the superposition of *U* acoustic source functions *f*_*u*_(*t*), where time-shifted versions of these source functions are linearly superposed with the mixing weights *a*_*ru*_. The time shifts are given by the time delays τ_*ru*_. This models the fact that for a reverberation-free room the signals from the acoustic sources arrive at the receiver with different time delays and attenuated amplitudes, which are dependent on the distances between the acoustic sources and the receivers. The corresponding generative model has the following form (for 1 ≤ *r* ≤ *R*):


(6) $$\begin{array}{l} y_{r}\left(t\right)=\overset{U}{\underset{u=1}{\sum }}a_{ru}\cdot f_{u}\left(t-\tau_{ru}\right)+residuals \end{array}$$

### 2.3. The anechoic mixture model subsumes previous modular models of movement generation

In this section, we show that all the models of spatial, temporal, and spatiotemporal modularity discussed so far can be considered as a special case of the anechoic mixture model (6).

#### 2.3.1. Derivation of the spatial decomposition model

Identifying the signals of type *y*_*r*_(*t*) with the components of the vectors **x**^*l*^(*t*), i.e., $y_{r}\left(t\right)=x_{m\left(r\right)}^{l\left(r\right)}\left(t\right)$ where the integer functions *l*(*r*) and *m*(*r*) define a one-to-one mapping between the *m*-th degree of freedom in trial *l* and the corresponding signal *y*_*r*_(*t*) (with 1 ≤ *r* ≤ *M* · *L*), and constraining the time delays τ_*ru*_ to be zero, one obtains the model (1). Since in this model the weight vectors **w**_*p*_ are assumed to be invariant over trials, all mixing weights *a*_*rp*_ belonging to the same DOF and primitive number *P* have to be equal and independent of the trial number, so that *a*_*rp*_ = *w*_*p,m*(*r*)_, where the function *m*(*r*) returns the number of the DOF that belongs to index *r* independent of the trial number. The time-dependent mixing coefficients $c_{p}^{l}\left(t\right)$ of the model (1) correspond to the source functions *f*_*u*_ of the model (6), thus $f_{u}\left(t\right)=c_{p\left(u\right)}^{l\left(u\right)}\left(t\right)$ where here the index *u* runs over all combinations of the indices *p* and *l*, thus 1 ≤ *u* ≤ *U* = *P* · *L* and where the integer functions *l*(*u*) and *p*(*u*) establish mappings between the number of the source function in model (6) and the time-dependent mixing weights in model (1). Non-negativity constraints can be added for the model parameters *a*_*rp*_and the functions *f*_*u*_(*t*), e.g., for the modeling of EMG data.

#### 2.3.2. Derivation of the temporal decomposition models

If one identifies the source functions in model (6) with the temporal primitive functions *s*_*p*_(*t*), i.e., *f*_*p*_(*t*) = *s*_*p*_(*t*), 1 ≤ *p* ≤ *P* and again constrains the delays τ_*ru*_ to be zero, Equation (6) becomes equivalent to model (2). In this case, the mixing weights *a*_*rp*_ are equated to the mixing coefficients $c_{mp}^{l}$ in model (2), where the index *r* runs over all combinations of *m* and *l*, formally $a_{rp}=c_{m\left(r\right),p}^{l\left(r\right)}$, with appropriately chosen integer functions *m*(*r*) and *l*(*r*). Like for model (1), the components of the data vector have to be remapped over DOF and trials according to the relationship $y_{r}\left(t\right)=x_{m\left(r\right)}^{l\left(r\right)}$(t). Again, non-negativity constraints can be added for the parameters *a*_*rp*_ and to the source functions *f*.

Additionally, assuming that the delays of the anechoic model can take on any value (τ_*ru*_ ≠ 0), and equating the delays in model (3) according to the relationship $\tau_{rp}=\tau_{m\left(r\right),p}^{l\left(r\right)}$, makes model (6) equivalent to model (3).

#### 2.3.3. Derivation of the spatiotemporal decomposition model

Introducing individual sets of basis functions for the different DOFs, grouping them into vectors and equating the mixing weights and temporal delays for the components of each vector, transforms model (6) into the model (4). On the level of the time-dependent basis functions, this equivalence can be mathematically described by the equation *f*_*u*_(*t*) = *w*_*p*(*u*), *m*(*u*)_(*t*), where *w*_*p,m*_ corresponds to the component of the basis function vector **w**_*p*_(*t*) that belongs to the *m*-th DOF, and where the integer functions *m*(*u*) and *p*(*u*) establish a one-to-one mapping between the indices of the basis functions in the two models and the number of the associated DOF. This assignment is independent of the trial index *l*. The index *r* in (6) runs over all combinations of DOF and trial numbers, thus 1 ≤ *r* ≤ *M* · *L*. The integer functions *m*(*r*) and *l*(*r*) assign the corresponding trial number and DOF to the index *r* in the model (6). Thus, the assignment equation for the data vector is again given by $y_{r}\left(t\right)=x_{m\left(r\right)}^{l\left(r\right)}\left(t\right)$ for the *m*-th DOF in the *l*-th trial. The requirement that all mixing weights and temporal delays belonging to the same basis function vector **w**_*p*_ are equal is equivalent to a set of equality constraints, which can be captured by the equation systems $a_{ru}=c_{p\left(r\right)}^{l\left(r\right)}$ and $\tau_{ru}=\tau_{p\left(r\right)}^{l\left(r\right)}$. Again, non-negativity constraints can be added, if necessary.

#### 2.3.4. Derivation of the space-by-time decomposition model

In order to establish equivalence with the model (5), the data vectors of the models are mapped onto each other according to the relationship $y_{r}\left(t\right)=x_{m\left(r\right)}^{l\left(r\right)}\left(t\right)$, where again *l*(*r*) and *m*(*r*) are integer mapping functions that assign the *r*-th element of the data vector of the model (6) to the *m*-th DOF of the data vector **x**^*l*^ for the *l*-th trial in (5) with 1 ≤ *r* ≤ *M* · *L*. Model (5) has a total of *P*_*sp*_ · *P*_*tp*_ temporal basis functions, where however the functional forms of the basis functions for different indices *q* (i.e., different spatial components) for the same *p* (i.e., same temporal component) just differ by time shifts. This is equivalent to an equality constraint for these functions, which can mathematically be characterized in the form *f*_*u*_(*t*) = *s*_*p*(*u*)_(*t*), with 1 ≤ *u* ≤ *P*_*tp*_ and the index functions *p*(*u*) and *q*(*u*) that map the index *u* in the model (6) onto the indices of the temporal and spatial primitive in (5). Since all indices with the same *p*(*u*) are mapped onto the same basis function *s*_*p*_, the last equation specifies an equality constraint. With the same integer mapping functions, finally, also the relationship between the mixing weights can be established, which is given by the equation $a_{ru}=c_{p\left(u\right),q\left(u\right)}^{l\left(r\right)}\cdot w_{q\left(r\right),m\left(u\right)}$, where *w*_*q,m*_ is the *m*-th element for the vector **w**_*q*_. The last equation specifies a bilinear constraint for the weight parameters of the model (6). Using the same notation, the equivalence between the delays is established by the equation system $\tau_{ru}=\tau_{p\left(u\right),q\left(u\right)}^{l\left(r\right)}$. A summary of the established equivalences between the general model (6) and the other models is given in Table 1.

**Table 1:** Constraints that make the primitive models (1), (2), (3), (4), and (5) equivalent to the general anechoic model (6). See text for details.

**Anechoic (6)**	**Spatial (1)**	**Temporal (2) or (3)**	**Spatiotemporal (4)**	**Space-by-time (5)**
$y_{r}\left(t\right)=\overset{U}{\underset{u=1}{\sum }}a_{ru}\cdot f_{u}\left(t-\tau_{ru}\right)$	${\text{x}}^{l}\left(t\right)=\overset{P}{\underset{p=1}{\sum }}{\text{w}}_{p}\cdot c_{p}^{l}\left(t\right)$	$x_{m}^{l}\left(t\right)=\overset{P}{\underset{p=1}{\sum }}c_{mp}^{l}\cdot s_{p}\left(t-\tau_{mp}^{l}\right)$	${\text{x}}^{l}\left(t\right)=\overset{P}{\underset{p=1}{\sum }}c_{p}^{l}\cdot {\text{w}}_{p}\left(t-\tau_{p}^{l}\right)$	${\text{x}}^{l}\left(t\right)=\overset{P_{tp}}{\underset{p=1}{\sum }}\overset{P_{sp}}{\underset{q=1}{\sum }}s\left(t-\tau_{pq}^{l}\right)\cdot c_{pq}^{l}\cdot {\text{w}}_{q}$
	$y_{r}\left(t\right)=x_{m\left(r\right)}^{l\left(r\right)}\left(t\right)$	$y_{r}\left(t\right)=x_{m\left(r\right)}^{l\left(r\right)}\left(t\right)$	$y_{r}\left(t\right)=x_{m\left(r\right)}^{l\left(r\right)}\left(t\right)$	$y_{r}\left(t\right)=x_{m\left(r\right)}^{l\left(r\right)}\left(t\right)$
	$f_{u}\left(t\right)=c_{p\left(u\right)}^{l\left(u\right)}\left(t\right)$	*f*_*p*_(*t*) = *s*_*p*_(*t*),	*f*_*u*_(*t*) = *w*_*p*(*u*), *m*(*u*)_(*t*)	*f*_*u*_(*t*) = *s*_*p*(*u*)_(*t*),
	*a*_*rp*_ = *w*_*p,m*(*r*)_	$a_{rp}=c_{m\left(r\right),p}^{l\left(r\right)}$	$a_{ru}=c_{p\left(r\right)}^{l\left(r\right)}$	$a_{ru}=c_{p\left(u\right),q\left(u\right)}^{l\left(r\right)}\cdot w_{q\left(r\right),m\left(u\right)}$
	τ_*ru*_ = 0	τ_*ru*_ = 0 or $\tau_{rp}=\tau_{m\left(r\right),p}^{l\left(r\right)}$	$\tau_{ru}=\tau_{p\left(r\right)}^{l\left(r\right)}$	$\tau_{ru}=\tau_{p\left(u\right),q\left(u\right)}^{l\left(r\right)}$

### 2.4. FADA: An efficient algorithm for the identification of motor primitives within the unified framework

All algorithms for blind source separation require the identification of a large number of parameters. For example, a complete definition of model (6) requires the specification of: *T* · *U* parameters to represent the sources *f*_*u*_(*t*), *R* · *U* parameters to represent the activation weights *a*_*ru*_, and *R* · *U* parameters to represent the delays τ_*ru*_. Thus, fitting a dataset with *L* trials and *M* degrees of freedom (i.e., with *R* = *M* · *L*), requires the estimation of a total of (*T* + 2*M* · *L*) · *U* parameters. In typical settings, the number of time samples *T* is much larger than the number of degrees of freedom *M*, sources *U*, and trials *L*. For applications in motor control, the relevant signals are subject to additional constraints, which can be exploited for the derivation of more efficient algorithms. Signals in motor control are typically smooth. This allows to reduce the complexity of the anechoic demixing problem considerably and to devise algorithms that are more robust than those developed for general purposes.

Here, we first present a robust algorithm for standard anechoic demixing, which can be used for the identification of the parameters associated with the unconstrained model (6). This algorithm, which relies on the representation of the signals in the frequency domain and is thus called Fourier-based Anechoic Demixing Algorithm (FADA), was introduced in a previous study (Chiovetto and Giese, 2013) to identify the temporal decomposition model (3). In the present work, we describe how this algorithm can be extended by inclusion of additional constraints to make it suitable for the identification of the parameters associated with all the most common models of motor modularity (1–5). The collection of algorithms for the estimation of all models of motor modularity is released as a public toolbox: the FADA toolbox (FADA-T, https://tinyurl.com/compsens).

The typical smoothness of the activity patterns (e.g., joint trajectories, EMG envelopes, etc.) recorded during the execution of body movements, implies that they can be well described by mixtures of smooth source functions (Chiovetto and Giese, 2013). These smooth source functions can, in turn, be well-approximated by truncated Fourier expansions, defined by only *K* nonzero complex Fourier coefficients, where *K* is typically far below the Nyquist limit (*K* ≪ *T*/2). Consequently, the number of parameters to identify to describe the original dataset drops to (*K* + 2*M* · *L*) · *U*. This decreases substantially the computational costs of the parameter estimation problem and makes it less prone to premature convergence to local minima.

Band-limited temporal signals *y*_*r*_(*t*) and source functions *f*_*u*_(*t*) can thus be approximated by truncated Fourier expansions of the form:


(7) $$\begin{array}{l} y_{r}\left(t\right)=\overset{K}{\underset{k=-K}{\sum }}c_{rk}e^{\frac{2\pi ikt}{T_{s}}} \end{array}$$

and


(8) $$\begin{array}{l} f_{u}\left(t-\tau_{ru}\right)≅\overset{K}{\underset{k=-K}{\sum }}\nu_{uk}e^{-ik\tau_{ru}}e^{\frac{2\pi ikt}{T_{s}}} \end{array}$$

where *c*_*rk*_ and ν_*uk*_ are complex constants (i.e., $c_{rk}=|c_{rk}|e^{i\varphi_{c_{rk}}}$ and $\nu_{uk}=|\nu_{uk}|e^{i\varphi_{\nu_{uk}}}$, where *i* is the imaginary unit, and φ_*c*_*rk*__ and φ_ν_*rk*__ are real numbers). The positive integer *K* is determined by Shannon's theorem according to the limit frequency of the signals, and *T*_*s*_ is the temporal duration of the signal. The source separation algorithm tries to ensure that the source functions *f*_*u*_(*t*) are uncorrelated over the distributions of the approximated signals. This implies $E\left\{f_{u}\left(t\right)\cdot f_{u^{\prime }}\left(t^{\prime }\right)\right\}=0$ for *u* ≠ *u*′ and any pair *t* ≠ *t*′. For the corresponding Fourier coefficients this implies $E\left\{\nu_{uk}\cdot \nu_{u^{\prime }k^{\prime }}\right\}=0$ for *u* ≠ *u*′ and any pair *k* ≠ *k*′. Combining Equations (6), (7), and (8) we obtain by comparison of the terms for the same frequency


(9) $$\begin{array}{l} c_{rk}=\overset{U}{\underset{u=1}{\sum }}a_{ru}\cdot \nu_{uk}e^{-ik\tau_{ru}} \end{array}$$

From this follows with $E\left\{\nu_{uk}\cdot \nu_{u^{\prime }k^{\prime }}^{*}\right\}=E\left\{|\nu_{uk}{|}^{2}\right\}\cdot \delta_{uu^{\prime }}$ the equation:


(10) $$\begin{array}{l} \left|c_{rk}{|}^{2}=E\{|c_{rk}|\right\}\\ \;=\overset{U}{\underset{u=1}{\sum^{\;}}}\sum_{u^{\prime }=1}^{U}a_{ru}a_{ru^{\prime }}E\{\nu_{uk}\cdot \nu_{u^{\prime }k^{\prime }}^{*}\}e^{-ik(\tau_{ru}-\tau_{ru^{\prime }})}\\ \;=\overset{U}{\underset{u=1}{\sum^{\;}}}a_{ru}^{2}E\{|\nu_{uk}{|}^{2}\}\\ \;=\overset{U}{\underset{u=1}{\sum^{\;}}}|a_{ru}{|}^{2}|\nu_{uk}{|}^{2} \end{array}$$

The symbol * indicates the conjugate of a complex number. The derivation of this equation replaces the expectations of the Fourier coefficients *c*_*rk*_ with their deterministic values and treats the source weights *a*_*rk*_ as deterministic trial-specific variables. This can be justified if these mixture weights are estimated separately from the sources in an EM-like procedure. Empirically, however, we obtain reasonable estimates of the model components based on Equation (10) also using other methods (see below). Since the signals *f*_*u*_(*t*) and *y*_*r*_(*t*) are real the corresponding Fourier coefficients fulfill $c_{rk}=c_{r,-k}^{*}$ and $\nu_{uk}=\nu_{u,-k}^{*}$. Thus, the demixing problem needs to be solved only for parameters with *k* ≥ 0.

The previous considerations motivate the following iterative algorithm for the identification of the unknown parameters in model (6). After random initialization of the parameters to be estimated, the following steps are carried out iteratively until convergence:

1. Compute the absolute values of the coefficients *c*_*rk*_ and solve the following equations:
  (11) $$\begin{array}{l} |c_{rk}{|}^{2}=\overset{U}{\underset{u=1}{\sum }}|a_{ru}{|}^{2}|\nu_{uk}{|}^{2} \end{array}$$
  with *r* = 0, 1, … *R* and *k* = 0, 1, … *K*. In our study we exploited non-negative ICA (Højen-Sørensen et al., 2002) to solve this equation. In the distributed version of the software, the Equation (10) can also be solved *via* non-negative matrix factorization (Lee and Seung, 1999, 2000).
2. Initialize for all pairs and iterate the following steps:
  (a) Update the phases of the Fourier coefficients of the sources, defined as φ_ν_*uk*__ = angle(ν_*uk*_) = arctan[Im(ν_*uk*_)/Re(ν_*uk*_)] by solving the following non-linear least square problem
    (12) $$\begin{array}{l} \underset{\Phi }{\text{min}}∥\text{C}-\text{Z}\left(\Phi \right){∥}_{F}^{2} \end{array}$$
    where (**C**)_*rk*_ = *c*_*rk*_, ${\left(\text{Z}\right)}_{rk}=\overset{U}{\underset{u=1}{\sum }}a_{ru}e^{-ik\tau_{uk}}|\nu_{uk}|e^{i\varphi_{\nu_{uk}}}$ and ∥∥_*F*_ indicates the Frobenius norm. In order to avoid cluttered notation, for the function **Z**(.) only the arguments with relevance for the optimization are explicitly written.
  (b) Keeping the identified source functions *f*_*u*_(*t*) constant, identify for each signal *y*_*r*_(*t*) the weights *a*_*ru*_ and delays τ_*ru*_ by minimization of the following cost functions:

(13) $$\begin{array}{l} \underset{{\text{a}}_{r},\tau_{r}}{\arg\min}∥y_{r}\left(t\right)-\text{f}{\left(t,\tau_{r}\right)}^{\prime }{\text{a}}_{r}{∥}_{F}^{2} \end{array}$$

where ′ is the transposition operator. Optimization with respect to **a**_*r*_ and **τ**_*r*_ is feasible, assuming uncorrelatedness of the functions *f*_*u*_ and independence of the time delays (Swindlehurst, 1998). The column vector **a**_*r*_ concatenates all weights related to DOF *r*, i.e., ${\text{a}}_{r}={\left[a_{r1},\ldots ,a_{rU}\right]}^{\prime }$. The vector function ${\text{f}}_{r}\left(t,\tau_{r}\right)={\left[f_{1}\left(t-\tau_{r1}\right),\ldots ,f_{U}\left(t-\tau_{rU}\right)\right]}^{\prime }$ concatenates the functions *f*_*u*_, shifted by the time delays related to the *r*-th DOF.

The original version of the FADA algorithm was designed to solve the source separation problems without constraints. Additional constraints, such as the non-negativity of the parameters or additional equality constraints for the weights and delays can be easily added, due to the modular structure of the algorithm. A detailed description of how we imposed such constraints can be found in the Appendix. Importantly, the addition of such constraints allowed us to develop a unifying method to identify the parameters of all the considered models of motor modularity.

### 2.5. Validation of the proposed framework

**Figure 1:**
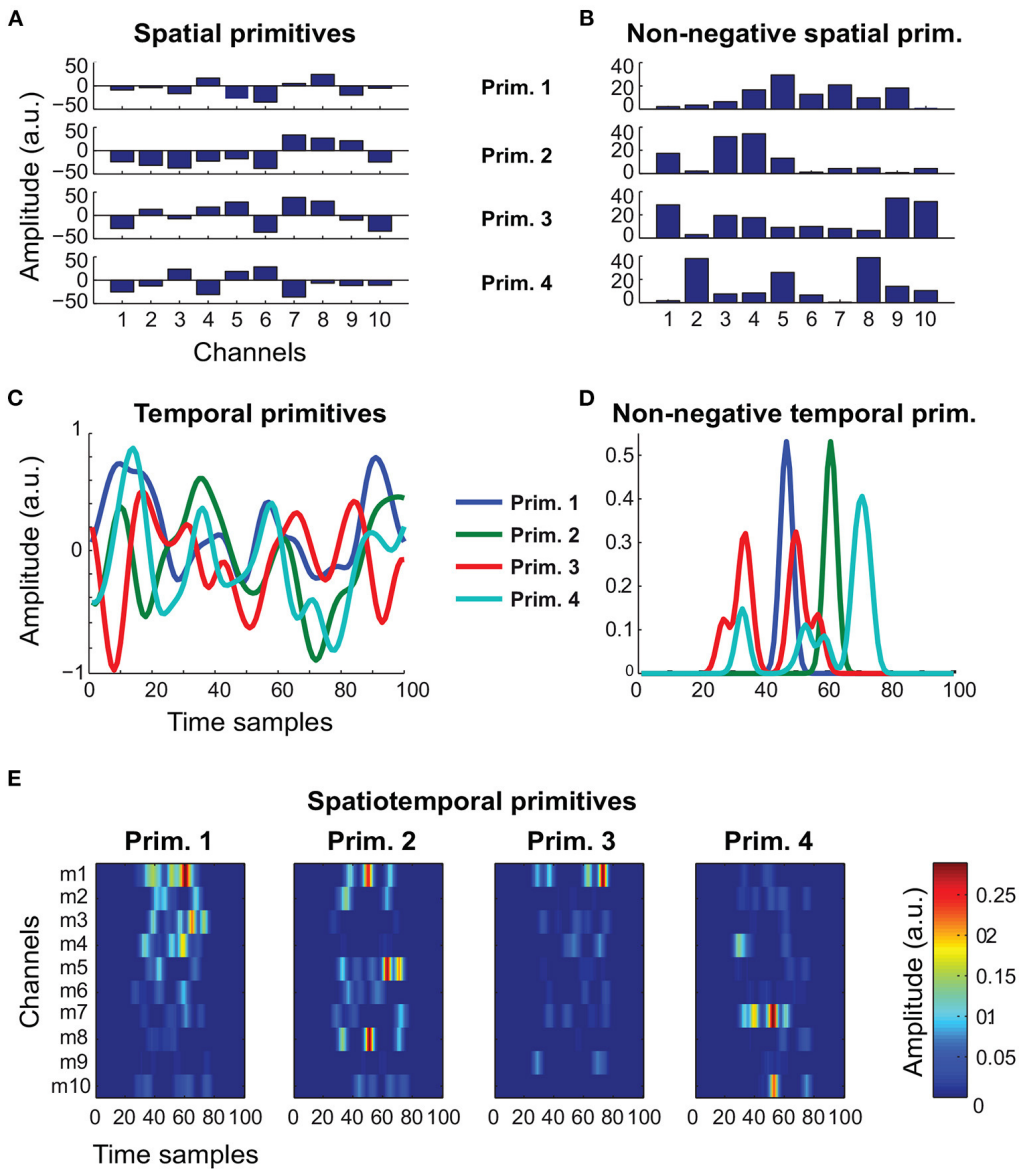
Representative primitives generated to assess the identification performance of FADA-T. **(A)** Unconstrained spatial primitives, associated with model (1). **(B)** Non-negative spatial primitives, associated with model (1) and (5). **(C)** Unconstrained temporal primitives, associated with models (2) and (3). **(D)** Non-negative (EMG-like) temporal primitives, associated with models (2), (3), and (5). **(E)** Spatiotemporal non-negative primitives, associated with model (4).

To validate our framework, we used both biologically realistic simulated data and real experimental data. The simulated data included a total of 20 noisy realization from the models (1–5), for five different noise levels. Importantly, for models (1–3), we generated data from both the unconstrained and the non-negative variants (Figure 1). This allowed us to simulate kinematic-like and EMG-like signals from a total of eight different models. We used the data generated from these models to benchmark the ability of FADA-T to identify the ground-truth generative models against that of other standard model-specific identification methods. These methods included: the fast Independent Component Analysis (fastICA—Hyvärinen and Oja, 1997), the Non-negative Matrix Factorization algorithm (NMF—Lee and Seung, 1999) the Anechoic Demixing algorithm (AnDem—Omlor and Giese, 2011), the Shifted ICA (SICA—Mørup et al., 2007b), the Anechoic NMF algorithm (AnNMF—Omlor and Giese, 2011), the shifted NMF (sNMF—Mørup et al., 2007a), the spatiotemporal NMF (stNMF—d'Avella et al., 2003), and the sample-based Non-negative Matrix tri-Factorization algorithm (sNM3F—Delis et al., 2014).

The experimental data included a dataset of body joint kinematics recorded during the execution of emotional walks (Omlor and Giese, 2007; Roether et al., 2009; Endres et al., 2013), and a dataset of arm EMG signals recorded during the execution of reaching movements (d'Avella et al., 2006). We fitted the temporal decomposition model with delays (3) to the kinematic dataset, and the spatiotemporal decomposition model (5) to the EMG dataset.

To assess the performance of the identification algorithms, we considered two measures. Specifically, as a measure of the ability of the algorithms to retrieve a solution consistent with the observed data, we computed the fraction of explained variance *R*^2^. As a measure of the ability of the algorithms to identify the ground-truth primitives, activation coefficients, and delays, we computed the normalized similarity *S*_*N*_ between the identified and ground-truth quantities. To normalize such measures, we computed a baseline similarity measure, which estimates the average similarity between random pairs of realizations of a single model. The normalized similarity *S*_*N*_ takes on values between zero and one, where zero indicates random estimates. Further details about the procedure we used to generate biologically realistic signals, the experimental dataset, the benchmark model-specific identification algorithms, and the similarity measures, can be found in the Appendix.

### 2.6. Statistical analyses

All tested measures were normally distributed according to a Chi-square goodness-of-fit test. Student's *t*-test was used to test whether the reconstruction and similarity measures were statistically different from the chance level. Group differences were statistically tested by two-way ANOVAs with Algorithm and Noise Level as factors. When appropriate, we performed *post-hoc* analysis with the Tukey-Kramer test. As a level of significance for the rejection of the null hypotheses we chose α = 0.05.

## 3. Results

### 3.1. Evaluation of algorithm performance on simulated data sets

To assess the identification performance of FADA-T, we generated EMG-like and kinematic-like ground-truth data, based on the mixture models defined by the Equations (1), (2), (3), (4), and (5). The aim of our comparison was to assess whether FADA-T could identify mixture parameters at least as well as other established model-specific unsupervised learning methods.

**Figure 2:**
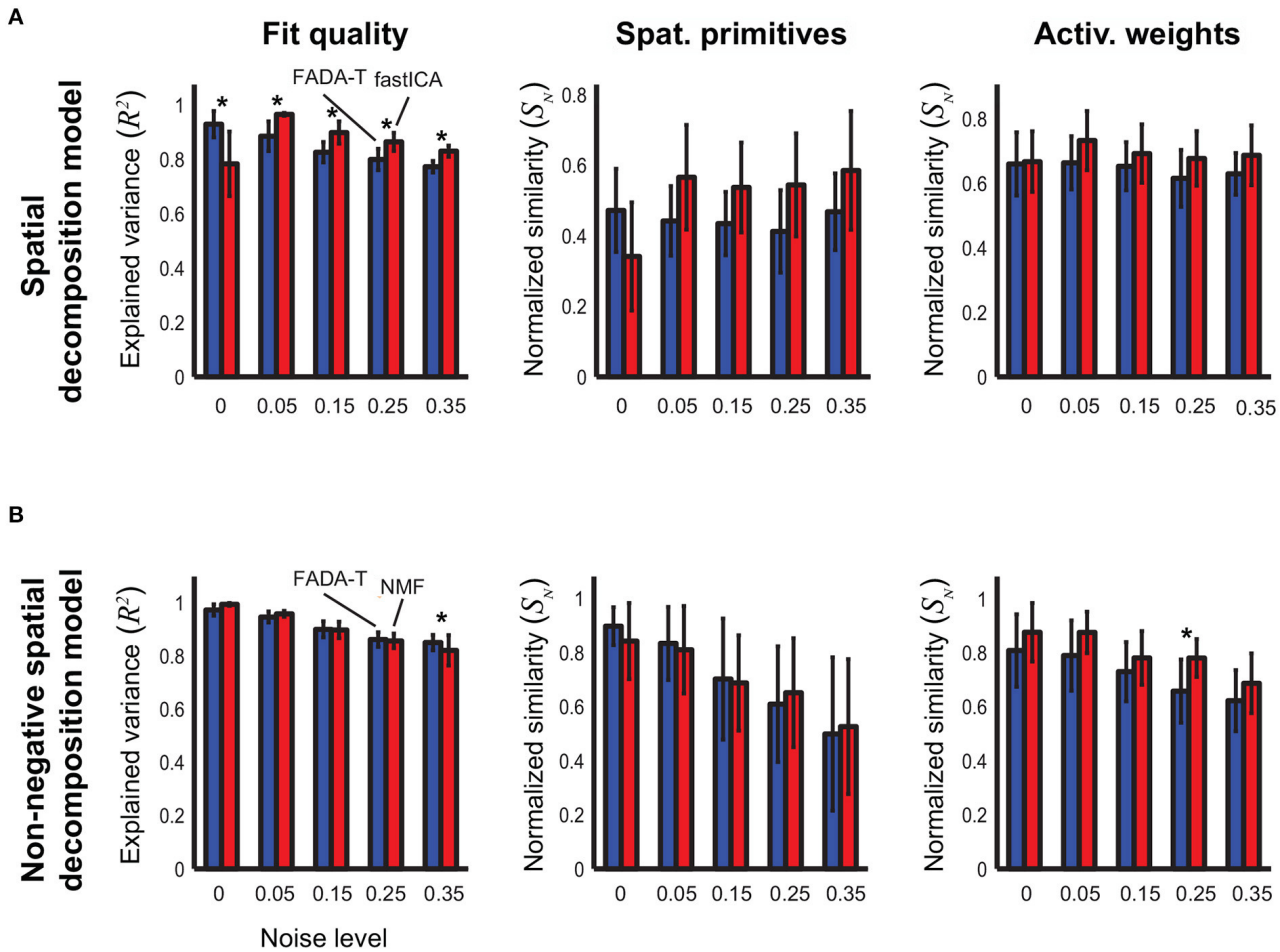
Identification of the spatial decomposition model. Performance (mean ± SD) of the Fourier-based Anechoic Demixing Algorithm Toolbox (FADA-T), fast Independent Component Analysis (factICA), and Non-negative Matrix Factorization (NMF) on artificial datasets generated with the spatial decomposition model and corrupted by increasing amounts of noise. **(A)** Unconstrained variant. From left to right: fraction of variance explained by the identified model, normalized similarities between original and identified primitives and activation weights. **(B)** Corresponding statistics for the model with positivity constraints. The * symbol indicates the statistically significant values of *p* < 0.05.

Figure 2A shows the average performance (±SD) of the FADA-T and the fastICA algorithms (Hyvärinen and Oja, 1997) on the identification of the parameters of the spatial decomposition model (1). The bar plots represent the reconstruction accuracy (*R*^2^) and the normalized similarity (*S*_*N*_) between the ground-truth and the extracted primitives and weights, averaged across 20 realizations, for five different levels of signal-dependent noise. Asterisks indicate significant differences between algorithms, according to *post-hoc* testing. Qualitatively, both algorithms provided a good level of reconstruction accuracy and resulted in an accurate estimation of the original model parameters. Accuracy measures were typically larger than 0.5, and the similarity measures for the recovered primitives and weighting coefficients were always significantly larger than chance [*t*_(19)_ > 9.93, *p* < 0.001]. The two-factor ANOVAs revealed a significant main effect of the Noise factor on both the reconstruction accuracy and the identification of the primitives [*F*_(4, 190)_ ≥ 5.08, *p* < 0.001], indicating a general decrease in performance for increasing levels of noise. Additionally, we found a significant main effect of the Algorithm factor on all the tested parameters [*F*_(1, 190)_ ≥ 11.92, *p* < 0.001]. The interaction between the two factors was also significant for the similarity of the primitives [*F*_(4, 190)_ ≥ 5.08, *p* < 0.001]. The *post-hoc* analysis revealed that the FADA and fastICA algorithms were equally able to retrieve the correct primitives and weights (*p* > 0.05), and that FADA-T was better able to reconstruct the simulated signals in the absence of noise (*p* < 0.05). However, fastICA tended to have higher reconstruction accuracy in the presence of noise (*p* < 0.05).

Figure 2B reports the identification performance of FADA-T on the spatial decomposition model (1) with non-negativity constraints. In this case, FADA-T was compared against the NMF algorithm (Lee and Seung, 1999), as fastICA does not provide a way to constrain parameters to be non-negative. Also in this case, both algorithms provided a good fit of the data and accurate estimates of the original primitives and mixture weights. Not surprisingly, performance of both algorithms degraded with increasing noise. ANOVAs indicated a significant main effect of the factor Algorithm on the similarity of the weighting coefficients [*F*_(1, 190)_ = 23.14, *p* < 0.001]. We also found a main effect of Noise Level on the *R*^2^ and *S*_*N*_ measures [*F*_(4, 190)_ ≥ 20.85, *p* < 0.001]. The interaction of both factors was significant only for the *R*^2^ measure [*F*_(4, 190)_ = 5.51,*p* < 0.001]. The *post-hoc* analysis showed that FADA-T had a higher reconstruction accuracy (*p* = 0.01) for the highest tested noise level (35%), and that FADA-T had a lower identification performance (*p* < 0.05) on the weight coefficients for the 25% noise level. Finally, all the measures were significantly above chance level [*t*_(19)_ ≥ 6.85, *p* < 0.001].

**Figure 3:**
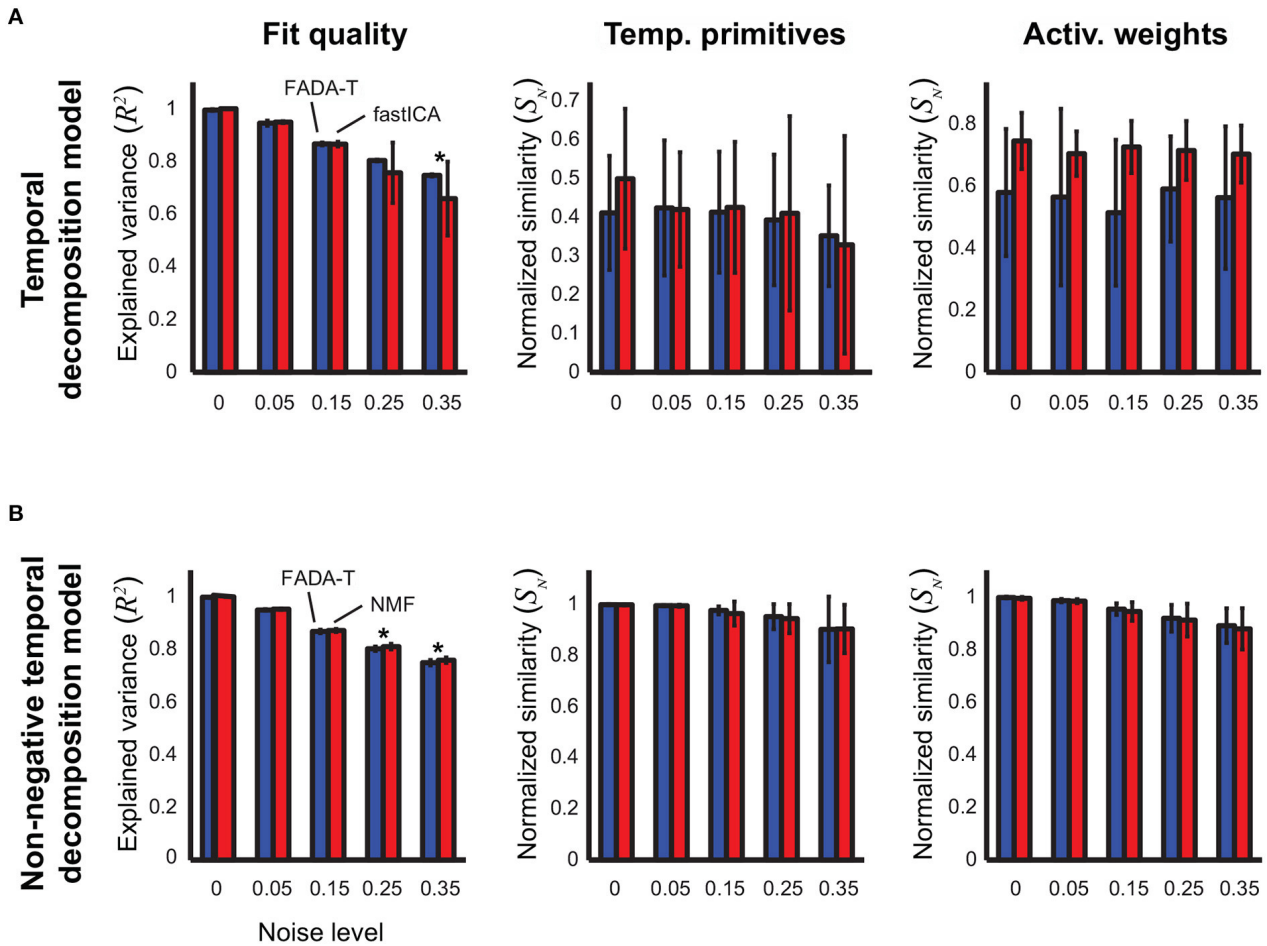
Identification of the temporal decomposition model (2). Performance of the FADA-T, factICA, and NMF algorithms on artificial datasets generated with the temporal decomposition model. **(A)** Unconstrained variant; from left to right: fraction of variance explained by the identified model, normalized similarities between original and identified primitives and activation weights. **(B)** Corresponding statistics for the model with positivity constraints. The * symbol indicates the statistically significant values of *p* < 0.05.

Figure 3A shows the identification performance of FADA-T and fastICA on the identification of the parameters of the temporal decomposition model without delays (2). Qualitatively, reconstruction accuracy and primitive similarity were modulated by the noise level, while weight similarity was not. ANOVAs confirmed this trend revealing a significant main effect of Noise on reconstruction accuracy and primitive similarity [*F*_(4, 190)_ ≥ 9.67, *p*<0.001]. The interaction between Algorithm and Noise Level was significant only for *R*^2^ [*F*_(4, 190)_ = 5.12, *p* < 0.001]. *Post-hoc* testing revealed that FADA-T and fastICA performed similarly (*p*>0.05), with the exception of the reconstruction quality for 35% noise level, where FADA-T outperformed fastICA.

Figure 3B shows the results of the comparison between FADA-T and NMF on the identification of the parameters of the temporal decomposition model without delays (2) with non-negativity constraints. The differences in performance between the two methods for the same noise levels were very small. Correspondingly, ANOVAs showed that the Algorithm factor had a significant main effect only on the reconstruction accuracy *R*^2^ [*F*_(1, 190)_ = 25.99, *p* < 0.001], while the Noise factor had significant main effects on all three tested measures [*F*_(4, 190)_ ≥ 17.38*p* < 0.001]. *Post-hoc* testing revealed that, for the two highest noise levels, the NMF algorithm approximated the original data with significantly higher reconstruction accuracy (*p* < 0.05). Finally, all measures in Figure 3 were significantly above chance level [*t*_(19)_ ≥ 8.86, *p* < 0.001].

Taken together, Figures 2, 3 show that, when applied to data generated with the synchronous models (1) and (2), FADA-T exhibited reconstruction performance overall comparable to those provided by the fastICA and NMF algorithms. In terms of the identification of the actual parameters, the differences between the tested algorithms were even smaller, with FADA-T underperforming only on the estimation of the weights of the constrained spatial decomposition model with 25% noise level.

**Figure 4:**
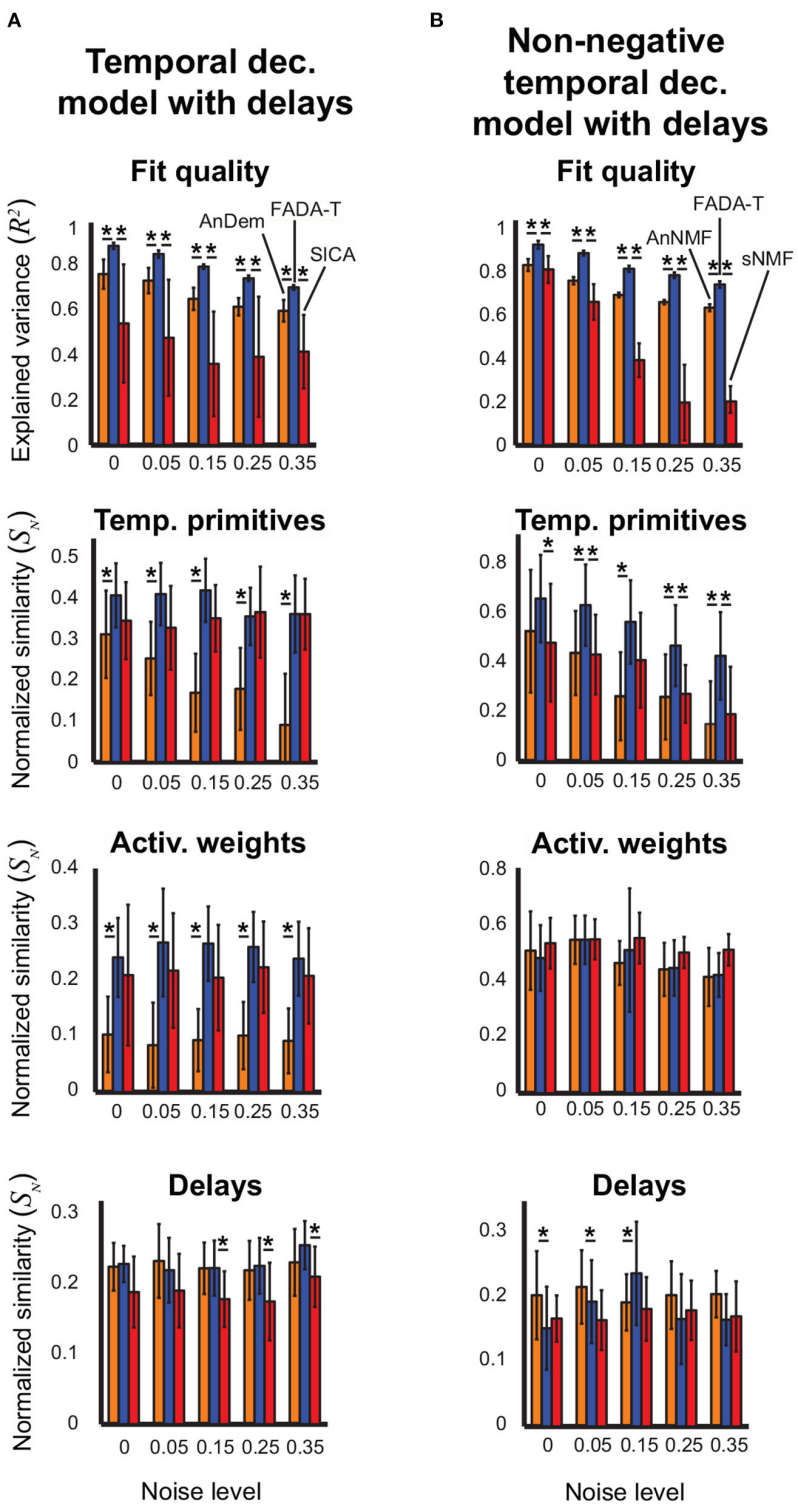
Identification of the temporal decomposition model with delays. Performance of FADA-T, Anechoic Demixing (AnDem), Shifted Independent Component Analysis (SICA), Anechoic Demixing with Non-negativity constraints (AnNMF), and shifted Non-negative Matrix Factorization (sNMF), on artificial datasets generated with the temporal decomposition model with delays. **(A)** Unconstrained variant. From top to bottom: fraction of variance explained by the identified model, normalized similarities between original and identified primitives, activation weights, and delays. **(B)** Corresponding statistics for the model with positivity constraints. The ** symbol represent two significant pairwise group differences (*p* < 0.05), each represented with a single asterisk (*).

In Figure 4, we show the performance of FADA-T on the identification of the temporal decomposition model with delays (3). For the unconstrained variant (Figure 4A), we compared FADA-T against the AnDem (Omlor and Giese, 2011) and the SICA (Mørup et al., 2007b) algorithms, while for the constrained variant (Figure 4B) we considered the AnNMF (Omlor and Giese, 2011) and the sNMF (Mørup et al., 2007a) algorithms. In addition to the similarity measures assessed for the models considered above, we also quantified the similarity between original and identified delays.

Figure 4A shows that, overall, FADA-T reconstructed the signals and identified the parameters of the unconstrained anechoic mixture better than the benchmark algorithms, for all the noise levels. ANOVAs revealed a significant main effect of the Algorithm factor on all the considered measures [*F*_(2, 190)_ ≥ 210.5, *p* < 0.001]. *Post-hoc* analysis revealed that, compared to AnDem, FADA-T provided significantly higher reconstruction accuracy, primitive similarity, and weight similarity (*p* < 0.05). Additionally, compared to SICA, FADA-T had a higher reconstruction accuracy for all noise levels (*p* < 0.001), and a higher delay similarity for noise levels >15% (*p* < 0.001). All measures in Figure 4A were significantly different from chance [*t*_(19)_ ≥ 3.23, *p* < 0.001].

Figure 4B shows that FADA-T tended to reconstruct the signals and identify the primitives of the non-negatives anechoic mixture model better than the benchmark algorithms, for all the noise levels. Specifically, we found a significant main effect of the factor Algorithm on *R*^2^, primitive similarity, and delay similarity [*F*_(2, 190)_ ≥ 6.64, *p* < 0.05]. *Post-hoc* testing revealed that, compared to the AnNMF, FADA-T had higher reconstruction accuracy across all noise levels (*p* < 0.001), a higher primitive similarity for the 5, 15, 25, and 35% noise levels (*p* < 0.05), and a higher delay similarity for the 15% noise level (*p* < 0.05). On the other hand, FADA-T had a lower delay similarity than AnNMF for the noise levels 0% and 5%. Compared to sNMF, FADA-T had higher reconstruction accuracy across all noise levels (*p* < 0.05), and higher primitive similarity for the 0, 5, 25, and 35% noise levels (*p* < 0.05). All similarity measures in Figure 4B were significantly above chance level [*t*_(19)_ ≥ 30.8, *p* < 0.01], except for the reconstruction accuracy provided by sNMF for the most noisy data sets [*t*_(19)_ = 0.25, *p* = 0.80].

**Figure 5:**
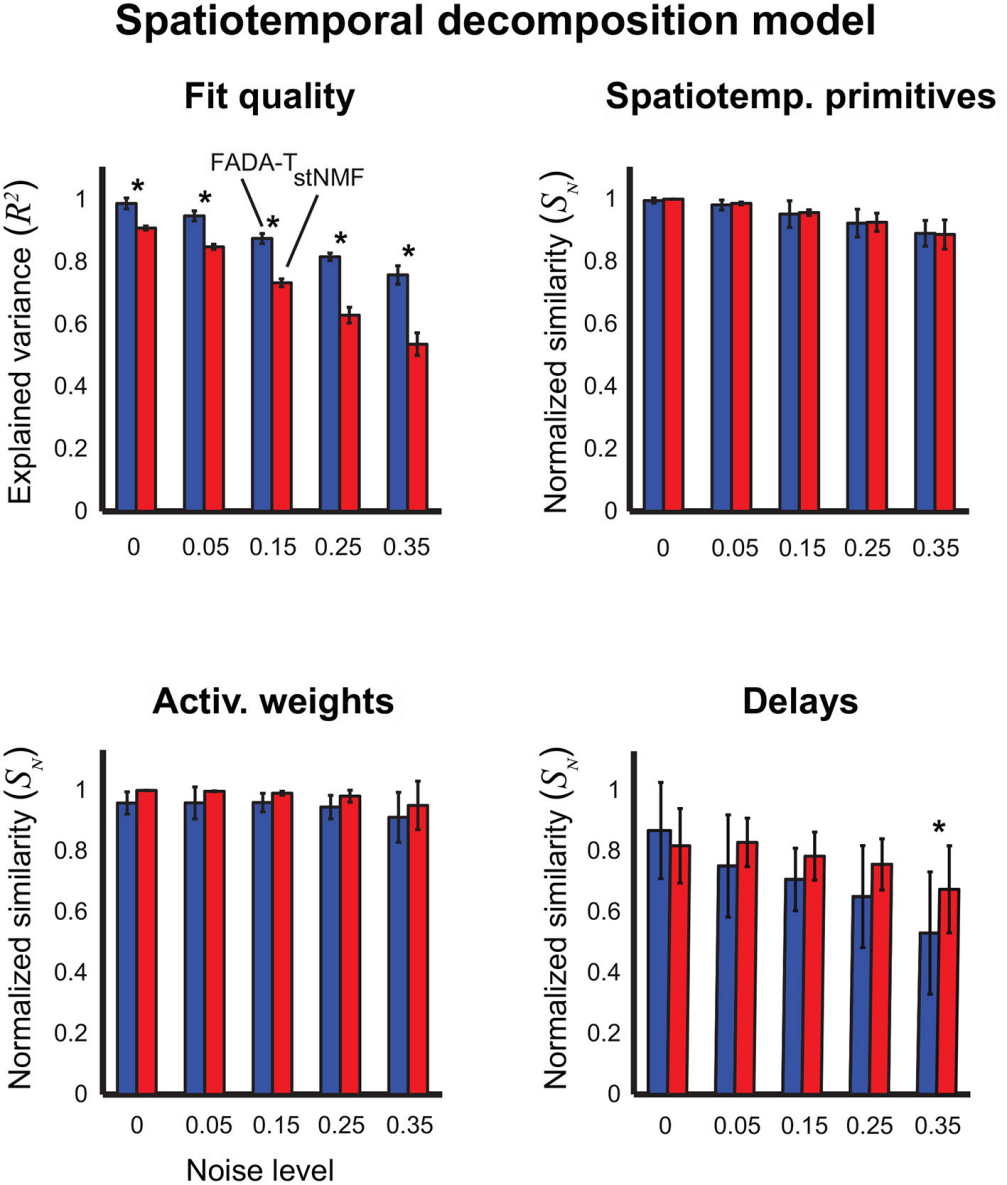
Identification of the spatiotemporal decomposition model (4). Performance of FADA-T and spatiotemporal NMF (stNMF) on artificial datasets generated with the spatiotemporal decomposition model. The (top) panels report the fraction of explained variance (left) and the normalized similarities between original and identified spatiotemporal primitives (right). The (bottom) panels report the normalized similarities between the corresponding activation weights (left) and delays (right). The * symbol indicates the statistically significant values of *p* < 0.05.

In Figure 5, we report the results on the identification of the spatiotemporal decomposition model (4). In this case, we compared FADA-T against the stNMF algorithm (d'Avella et al., 2003), developed specifically to fit this model. In this case, we found a significant main effect of the Algorithm factor on the reconstruction accuracy, weight similarity, and delay similarity [*F*_(1, 190)_ ≥ 13.34, *p* < 0.001], but not on the primitive similarity [*F*_(1, 190)_ = 0.4, *p*>0.05]. We also found a significant Algorithm by Noise interaction on reconstruction quality and delay similarity [*F*_(4, 190)_ ≥ 2.84, *p* < 0.05]. *Post-hoc* testing revealed that FADA-T had a higher reconstruction accuracy than stNMF across all noise levels (*p* < 0.001); however, FADA-T had a lower delay similarity for the 35% noise level (*p* = 0.03). Finally, all the tested measures were significantly above chance level for all the noise levels [*t*_(19)_ ≥ 11.78, *p* < 0.001].

**Figure 6:**
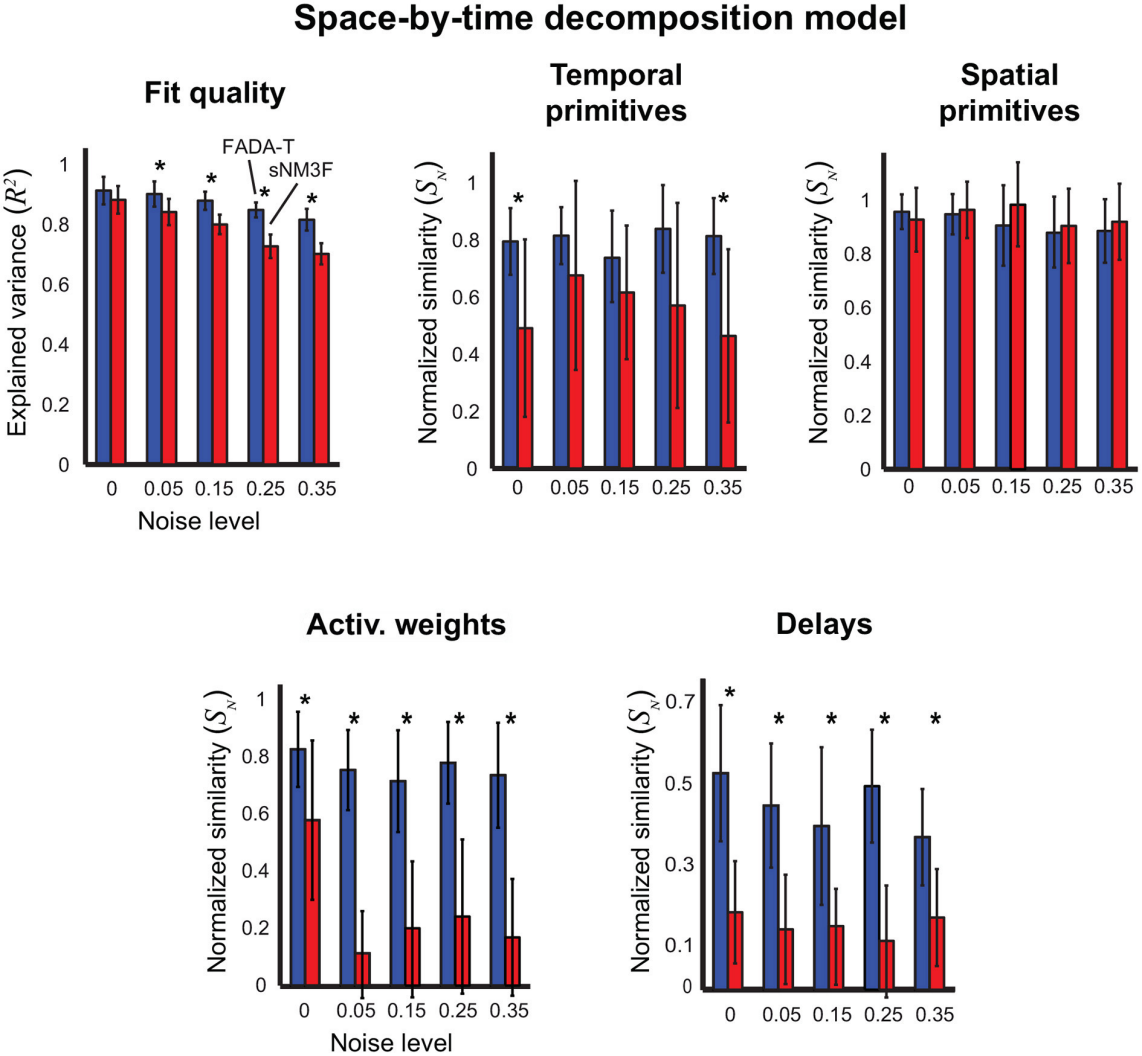
Identification of the space-by-time decomposition model. Performance of FADA-T and the sample-based Non-negative Matrix tri-Factorization (sNM3F) algorithm on artificial datasets generated with the space-by-time decomposition model. The (top) panels report the fraction of explained variance (left) and the normalized similarities between ground-truth and estimated temporal (center) and spatial (right) primitives. The (bottom) panels report the normalized similarities between the corresponding activation weights (left) and delays (right). The * symbol indicates the statistically significant values of *p* < 0.05.

Figure 6 summarizes the results on the identification of the space-by-time decomposition model (5). In this case, we compared FADA-T against the sNM3F algorithm (Delis et al., 2014), which was developed to fit this specific model. Qualitatively, FADA-T appears to perform better than sNM3F, especially in terms of reconstruction quality, weight similarity and delay similarity. The statistical analyses revealed: a main effect of the Algorithm factor on all the variables [*F*_(1, 190)_ ≥ 10.72, *p* < 0.001]; a main effect of Noise on reconstruction accuracy, weight similarity, and delay similarity [*F*_(4, 190)_ ≥ 2.74, *p* < 0.05]; a significant Algorithm by Noise interaction on reconstruction accuracy, spatial primitive similarity, and weight similarity [*F*_(4, 190)_ ≥ 2.46, *p* < 0.05]. *Post-hoc* testing showed that, in the presence of noise, FADA-T had a significantly better reconstruction accuracy than sNM3F (*p* < 0.001). Additionally, FADA-T had a higher temporal primitive similarity for the noise levels 0 and 35% (*p* < 0.01), and a comparable spatial primitive similarity (*p* > 0.05). Moreover, FADA-T outperformed sNM3F on the estimation of weights and delays, across all noise levels (*p* < 0.05). Finally, *t*-tests showed that FADA-T was always able to provide above chance estimates [*t*_(19)_ ≥ 3.68, *p* < 0.01], while sNM3F provided chance-level weight estimates for the 5% noise level [*t*_(19)_ = 1.91, *p* = 0.07].

In summary, these results seem to indicate that, on simulated data, FADA-T performs comparably well to model-specific methods on the identification of synchronous mixture models (Figures 2, 3) and better on the identification of anechoic mixtures (Figures 4–6).

### 3.2. Evaluation of algorithm performance on real experimental data

**Figure 7:**
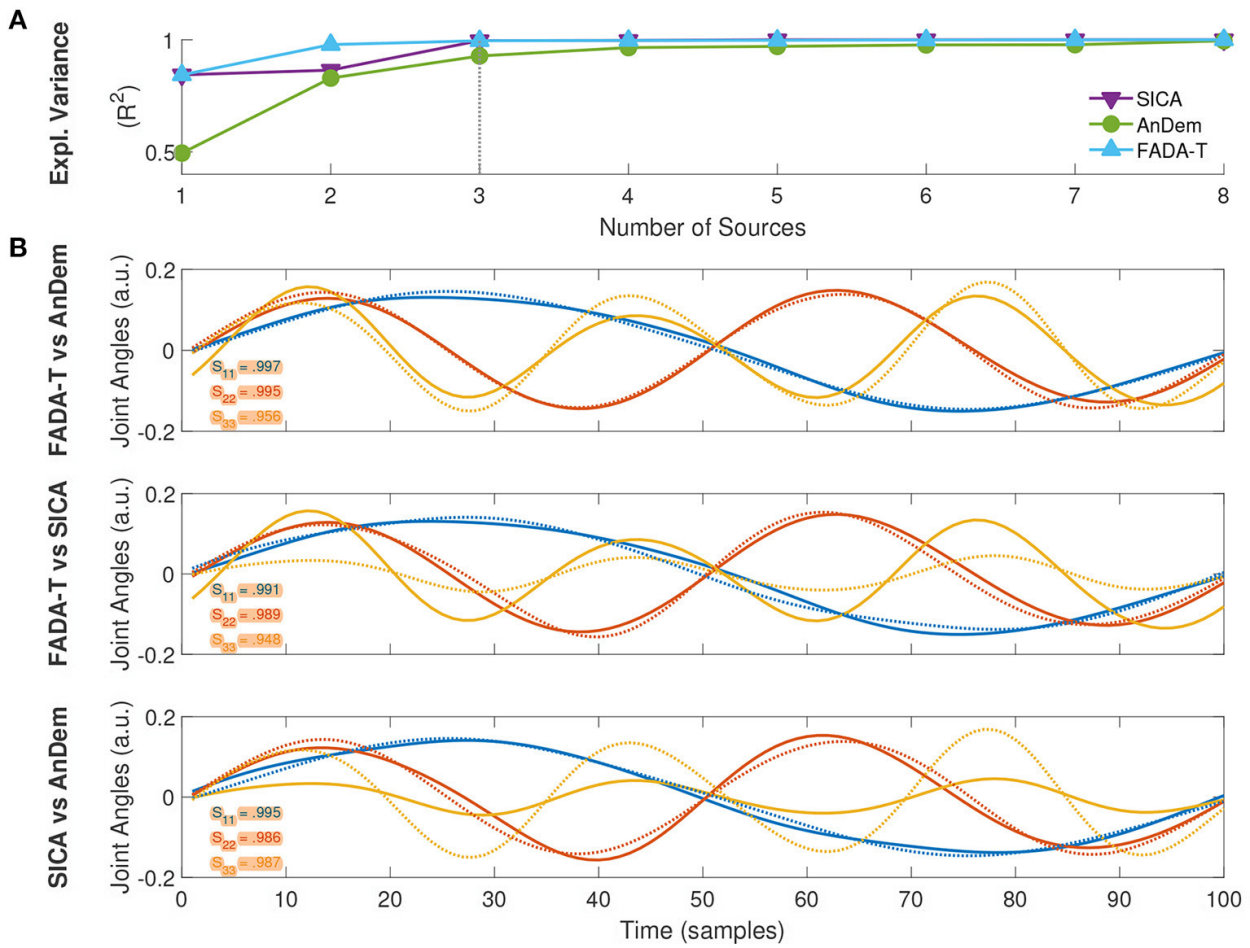
Extraction of delayed temporal primitives from kinematic data of emotional walking. **(A)** Fraction of explained variance as a function of the number of extracted primitives, identified with FADA-T (cyan), AnDem (green), and SICA (purple). **(B)** Temporal primitives identified by the three algorithms for a model with three sources; legends indicate pairwise correlation values.

In addition to validating FADA-T on synthesized data, we also assessed its performance on previously published real experimental data, comparing the primitives extracted by FADA-T with those identified with other techniques. The first experimental data set consisted of kinematic joint angle trajectories of the body joints of participants performing emotional walks. Trajectories represented a single gait cycle, resampled with 100 time steps (Roether et al., 2009; Endres et al., 2013). In this case, we tested FADA-T against the AnDem (Omlor and Giese, 2011) and the SICA (Mørup et al., 2007b) algorithms on the extraction of delayed temporal primitives (3). Figure 7A shows the fraction of explained variance (*R*^2^), as a function of the number of primitives. Consistently with previous studies (i.e., d'Avella et al., 2006; Berger et al., 2020), to select the number of primitives that allow a good trade-off between reconstruction accuracy and model complexity, we used the *elbow method*: we selected the minimum number of primitives from which a line could fit the *R*^2^ curve well (i.e., with a mean squared error below 10^−4^—d'Avella et al., 2006; Berger et al., 2020). For all methods, this point was reached for *N* = 3, indicating that three anechoic components (Figure 7B) can approximate the experimental data set well. Interestingly, the primitives identified by the methods were almost identical (*S* ≥ 0.94).

**Figure 8:**
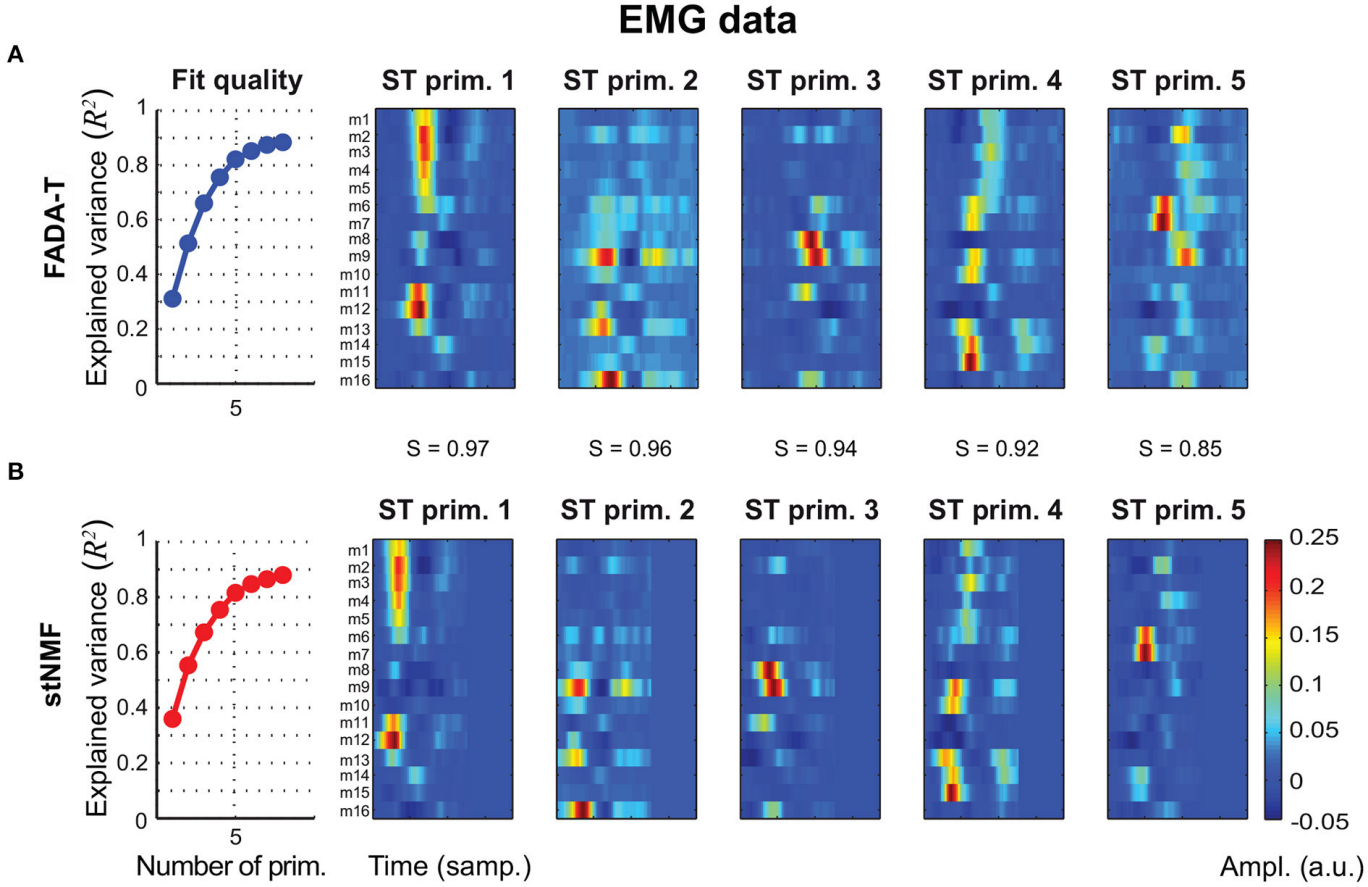
Extraction of spatiotemporal primitives from EMG data during reaching movements. Primitives are extracted with the FADA-T **(A)** and stNMF **(B)**, and are grouped according to their similarity. The leftmost panels show the fraction of explained variance as function of the number of extracted primitives. The remaining panels show the activation patterns of the spatiotemporal primitives extracted by the two algorithms.

The second experimental dataset we used to validate FADA-T comprised EMG signals recorded from 16 different task-relevalant muscles during point-to-point arm reaching movements (d'Avella et al., 2006). In this case, we fitted the spatio-temporal decomposition model (4)—which was originally shown to capture important features of the dataset (d'Avella et al., 2006)—with both FADA-T and the stNMF algorithm. Figure 8 shows that the methods displayed very similar *R*^2^ curves (first column), which leveled off at *N* = 5 (as assessed by the elbow method). Additionally, also in this case, the identified spatiotemporal primitives identified by the two methods were very similar (*S* ≥ 0.85).

Taken together, these results suggest that FADA-T performs as well as model-specific identification methods on both real kinematic (Figure 7) and muscle activity data (Figure 8).

## 4. Discussion

In this work, we have introduced a novel framework that allows to unify a number of common methods for the definition and identification of spatial, temporal, and spatiotemporal motor primitives. The framework harnesses the flexibility of the anechoic mixture model to capture qualitatively different classes of motor modularity models, and the robustness of the Fourier-based Anechoic Demixing Algorithm (FADA) to estimate the parameters reliably. We tested the framework on eight different model classes on both simulated and experimental data and showed that the reconstruction and identification performance were in most cases comparable to those achieved by established model-specific identification methods. As the experimental data comprised both electromyographic and kinematic spatio-temporal signals collected during the execution of qualitatively different motor behaviors, our results suggest that the framework constitutes a valid unsupervised method to identify spatial, temporal, and spatiotemporal regularities from signals extracted from different levels of the motor hierarchy. Our framework has thus the potential to facilitate the identification of motor modules, and to remove the potential confounding arising when comparing results obtained adopting different models and identification algorithms.

The algorithmic framework we used to identify the sources, delays, and mixing weights of the anechoic mixture model builds on the FADA algorithm, which we originally introduced in Chiovetto and Giese (2013) for the estimation of the temporal decomposition model with delays (3). In the present work, we have extended FADA to make it suitable for the identification of other popular models of spatial and spatiotemporal decomposition. This was accomplished by deriving classes of relevant constraints and by appropriately adapting individual optimization steps. The resulting classes of algorithms, which we have released publicly in the FADA toolbox (FADA-T), solve the well-known problem of over-determined anechoic demixing, where the number of signals to reconstruct outnumbers that of the latent source functions. A few algorithms have been proposes to address such a problem, including the Shifted Factor Analysis (Harshman et al., 2003), the Shifted Independent Component Analysis (Mørup et al., 2007b), and the Anechoic Demixing Algorithm (Omlor and Giese, 2011). However, these algorithms tend to be computationally expensive, as they do not sufficiently restrict the search space of the latent sources. Our identification framework, on the other hand, imposes a smoothness prior on the sources; this restricts the search space to band-limited source function, which speeds up the estimation process.

Influential studies have compared the performance of different existing identification methods for the estimation of motor primitives (e.g., Tresch et al., 2006; Lambert-Shirzad and Van der Loos, 2017; Ebied et al., 2018), or for the blind source decomposition of other biological signals (Virtanen et al., 2009; Cashero and Anderson, 2011; Erhardt et al., 2011). In contrast to these studies, here we have introduced a novel framework for the identification of motor primitives; moreover, we have shown that the framework allows the estimation of several classes of motor primitives and have benchmarked it against popular model-specific identification methods. Similarly to these studies, we have used both simulated and real experimental data to measure the identification performance.

A key element of the FADA algorithm is the mapping onto a finite Fourier basis. On the one hand, this strategy reduces remarkably the number of identified parameters in comparison to more general anechoic demixing methods (Mørup et al., 2007b; Omlor and Giese, 2011). On the other hand, this choice determines that only band-limited data can be adequately modeled. However, this is usually not an issue of major concern in fields such as motor control, where the typical activity patterns of interest (e.g., joint kinematics, EMGs, etc.) are naturally band-limited or artificially smoothed by filtering and trial averaging. An additional potential limitation of our work is that the current implementation of FADA-T includes some constrained optimization steps performed with gradient descent methods, which can potentially be time-consuming. Even though FADA-T performs such steps relying on built-in MATLAB functions, future work can potentially replace them with more efficient ones, harnessing the highly modular pipeline of FADA-T.

Empirically, we found that the imposed reduction of the dimensionality of the parameter space resulted in a more robust estimation of the primitives (even in the presence of substantial noise levels) and in a lower probability of convergence to local minima. Consequently, FADA-T performed better than other methods on the identification of anechoic mixture models (Figures 4–6). This seems to be due to a stronger ability to identify the correct weights and delays, especially in the cases of the space-by-time decomposition model (Figure 6), and the temporal decomposition model with delays (Figure 4A). Preliminary analyses, also suggest a better ability of FADA-T to deal with ambiguities in the estimation of delays and source functions, especially for sources with higher fundamental frequencies. Further studies, which are beyond the scope of this paper, will investigate this issue in more depth.

The only case where FADA-T showed consistently lower performance was on the identification of the unconstrained spatial decomposition model (Figure 2A), where fastICA had higher reconstruction accuracy in the presence of noise. We speculate that this happened because the smoothness prior imposed by FADA-T on the spatial domain was perhaps too restrictive. However, in spite of this problem, both the reconstruction and identification accuracy were sufficiently high. In addition to testing FADA-T on simulated datasets, we also considered the problem of identifying spatiotemporal sources from real experimental data of EMG and kinematic data sets, collected from human participants during the execution of goal-oriented and rhythmic motor tasks. In this case, our results show that FADA-T retrieved sources and mixing coefficients that were consistent with those obtained with other traditional techniques (cf. Figures 7, 8).

The central mathematical contribution of this article is the systematical analysis of the relationship between the different models of motor modularity, and, most importantly, their common derivation from the anechoic mixture model (6). This raises the question of how to identify the most appropriate modularity model for a given experimental dataset. Even though we have not proposed new solutions to this problem in this work, classical model selection methods can easily be applied to this context, including the Akaike method (Akaike, 1974) and the Bayesian Information Criterion (Schwarz, 1978). Alternative approaches such as Bayesian model selection (Bishop and Nasrabadi, 2006) can also be potentially applied. For this purpose, all tested models are embedded in a joint model space, and one marginalizes the prediction error (evidence) using an uninformative prior distribution over all possible model architectures. This procedure typically finds automatically a good balance between the goodness-of-fit and simplicity of the model. We have proposed an implementation of this idea for automatic model selection in Endres et al. (2013), where we approximated the resulting non-Gaussian distributions with a Laplace approximation. This allowed us to obtain an analytically tractable criterion to compare different demixing models, including those with time delays. Interestingly, a similar procedure can also allow to make inference about the most suitable smoothness priors for a given data set. A more recent AIC-based approach for the estimation of the number of motor primitives extracted with NMF is introduced by Ranaldi et al. (2021).

## 5. Conclusion

Experimental investigations over the last couple of decades have confirmed Bernstein's hypothesis (Bernstein, 1966) that the CNS simplifies the control of movement by relying on a modular organization of control (Flash and Hochner, 2005; Bizzi et al., 2008; Bizzi and Ajemian, 2020). The modules underlying such a control architecture have been defined in multiple ways (Tresch et al., 2006; Chiovetto et al., 2013; Giszter, 2015), and extracted by applying a variety of unsupervised learning algorithms to kinematic, kinetic, EMG, and neural data. In this work, we have introduced a unifying framework as a potential solution to this heterogeneity of approaches. FADA-T—the toolbox we have developed to provide a single estimation environment for the most common models of motor modularity—promises to facilitate the interpretation of the multimodal data recorded during the execution of body movements, by simplifying the process of identifying the most appropriate modularity models for the dataset at hand. We expect FADA-T to also stimulate the explorative adoption of the motor modularity models to other neuroscientific domains, which can potentially lead to the discovery of similar principles of hierarchical organization in other functional brain systems.

## Data availability statement

The datasets analyzed in this study are available from the corresponding author on reasonable request.

## Ethics statement

Ethical review and approval was not required for the study on human participants in accordance with the local legislation and institutional requirements. Written informed consent from the patients/participants or patients/participants' legal guardian/next of kin was not required to participate in this study in accordance with the national legislation and the institutional requirements.

## Author contributions

EC, AS, Ad'A, and MG have made a significant contribution to the design of the study, the development and implementation of the methods, the analysis of the results, and the writing of the manuscript. All authors contributed to the article and approved the submitted version.

## Funding

This work was supported by the German Federal Ministry of Education and Research (BMBF FKZ 01GQ1704), the Human Frontiers Science Program (HFSP RGP0036/2016), the German Research Foundation (DFG GZ: KA 1258/15- 1), the European Research Council (ERC 2019-SyGRELEVANCE- 856495), and the European Union Horizon 2020 Programme (CogIMon H2020 ICT-23-2014/644727).

## Conflict of interest

The authors declare that the research was conducted in the absence of any commercial or financial relationships that could be construed as a potential conflict of interest.

## Publisher's note

All claims expressed in this article are solely those of the authors and do not necessarily represent those of their affiliated organizations, or those of the publisher, the editors and the reviewers. Any product that may be evaluated in this article, or claim that may be made by its manufacturer, is not guaranteed or endorsed by the publisher.
