# Supplementary material for: Toward a unifying framework for the modeling and identification of motor primitives
Source: Front Comput Neurosci. 2022 Sep 12;16:926345. doi: 10.3389/fncom.2022.926345 (PMC9510628; doi:10.3389/fncom.2022.926345)
Supplement: Supplementary file 1 [file Data_Sheet_1.pdf]

APPENDIX

A DEALING WITH MODEL-SPECIFIC CONSTRAINTS

A.1 Estimation of non-negative source functions

For the case where the primitives f_u can assume only non-negative values, equation (10) cannot be derived in the way discussed above, and the expression of the non-negativity constraints in the Fourier representation is not straightforward. We decided, instead to estimate the time-dependent values of $f_u(t)$ directly, taking the inequality constraint $f_u(t) \geq 0$ for discretely sampled values of into account. This results in the following algorithm: Starting from random values of the parameters, the following three steps are iterated until convergence:

1. Update of the absolute values of the Fourier coefficients $|\nu|_{uk}$ of the primitives f_u, assuming their phases $\varphi_{\nu_{uk}}$and the mixing weights a_{ru} are known, by solving the non-linear constrained optimization problem:

$$\begin{aligned} &\underset{\mathbf{N}}{\text{minimize}} && \|\mathbf{C} - \mathbf{Z}(\mathbf{N})\|_F^2 \\ &\text{subject to} && f_u(\mathbf{N}, t) \geq 0, \; u = 1, 2, \dots U \text{ and } t = 1, \dots T. \end{aligned} \tag{14}$$

Note that, in order to avoid cluttered notation, for the functions $\mathbf{Z}(.)$ and $f_u(.)$ only the arguments with relevance for the optimization are explicitly written. The matrix is defined as in (12) and

$$(\mathbf{Z})_{rk} = \sum_{u=1}^{U} a_{ru} e^{-ik\tau_{uk}} |\nu_{uk}| \, e^{i\varphi_{\nu_{uk}}} \tag{15}$$

with $(\mathbf{N})_{uk} = |\nu_{uk}|$.

2. Keeping the other parameters fixed, update the phases $\varphi_{\nu_{uk}}$ of the Fourier coefficients of the primitives by solving the non-linear constrained optimization problem

$$\begin{aligned} &\underset{\mathbf{\Phi}}{\text{minimize}} && \|\mathbf{C} - \mathbf{Z}(\mathbf{\Phi})\|_F^2 \\ &\text{subject to} && f_u(\mathbf{\Phi}, t) \geq 0, \; u = 1, 2, \dots U \text{ and } t = 1, \dots T. \end{aligned} \tag{16}$$

Remind that the Fourier coefficients ν_{u0} are real so that it is sufficient to regard consider only $k = 1, \dots, K$.

3. Update weights and delays as in the unconstrained version of FADA by solving the optimization problem (13).

A.2 Estimation of non-negative mixing coefficients

Non-negativity of the scaling coefficients a_{ru} of the primitives can be easily imposed in the algorithm. In (13) the scaling coefficients are determined, assuming that primitives and temporal delays are known, solving a least squares problem. The same optimization problem can be solved adding the linear inequality constrains $a_{ru} \geq 0$, $\forall r, u$, resulting in a non-negative least squares problem for the weights a_{ru}.

A.3 Estimation of spatial primitives

The FADA algorithm presented above can be used to identify not only temporal, but also spatial primitives. This can be achieved simply by transposing the data matrix $\mathbf{X}$ and constraining all the delays in the algorithm to be equal to 0. In this way indeed, the FADA algorithm identifies a set of invariant spatial (instead of temporal) vectors, interpreting the elements of each vector $\mathbf{x}(t)$ as a series of time points.

Although there is no theoretical evidence for the existence of any smoothness relation between the values of the different DOF at a given time instant t (so that the smoothness assumptions of FADA on the data are satisfied), it will be shown in the next sections how the algorithm can however still provide identification performance at least as good as those associated with other standard machine learning techniques.

A.4 Estimation of spatiotemporal primitives

For the identification of spatiotemporal synergies, constraints for the parameters have to be set according to model (4). In a first step, for each DOF m in the data set P source functions f_p are assigned, resulting in a total of $M \cdot P$ independent source functions. The following three steps are then carried out iteratively until convergence:

1. The optimal delays τ_p^l for each spatiotemporal primitive are found, for each trial l, applying a matching pursuit procedure (Mallat and Zhang, 1993; d'Avella and Bizzi, 2005), consisting of an iterative search for a set of time-shifted primitives that best match the data. For each primitive, the scalar product between the original data and the time-shifted primitive is computed, testing all possible time delays between 0 and T-1. The primitive and delay associated with the highest scalar product is then selected and its contribution is subtracted from the data. Then the same procedure is repeated for the remaining primitives on the residual of the data. This search is repeated until all delays have been determined.
2. The combination coefficients c_p^l are updated by minimizing, for each trial l, the difference between the original data and the reconstruction, estimated exploiting model (4) and assuming that the source functions f_u and the delays τ_{ru} are known.
3. Assuming that the weights and the delays are known from the previous steps, the functions f_u, which correspond to the components of the spatiotemporal primitives $w_p(t)$ are updated. The Fourier coefficients of the corresponding source function are determined in the same fashion as for the original FADA algorithm without constrains. Non-negativity constraints for the primitives and weights can be imposed in the same way as described above.

A.5 Estimation of space-by-time primitives

To identify the space-by-time decomposition model, we exploited the core of FADA algorithm (the mapping onto the Fourier space) for the identification of the temporal primitives. Similarly to Delis and colleagues (Delis et al., 2014), our algorithm was also designed for the processing of EMG-like data and all the parameters in model (5) (with the exception of the delays) are constrained to be non-negative. Given the data matrix $\mathbf{X}$, in the first step of the algorithm P_{sp} spatial primitives $\mathbf{w}_q$ are identified, applying non-negative matrix factorization (Lee and Seung, 2000). Then the FADA algorithm is applied to $\mathbf{X}$ in order to identify P_{tp} non-negative temporal primitives $s_p(t)$. In the second step of the algorithm, the spatial primitives are kept constant, while temporal primitives, weights and delays are updated. The algorithm consists of the iteration of the two following steps:

1. The Fourier coefficients of the functions $s_p(t)$ are updated as in the constrained FADA algorithm, by minimizing the difference between the Fourier coefficients of the original data and the linear combination of the corresponding Fourier coefficients.
2. Weights and delays are updated minimizing the difference between the original data and the estimates provided by model (5). The optimal delays τ_{qp}^l are found for each trial l, following a matching pursuit procedure. Similarly, the weights c_{qp}^l are identified, solving for each trial a constrained linear least-squares problem.

B RELATED WORK ON ANECHOIC DEMIXING

The classes of algorithms we have developed to fit common models of motor modularity, publicly released in the FADA toolbox (FADA-T), solve the well-known problem of over-determined anechoic demixing, where the number of signals to reconstruct outnumbers that of the latent source functions. Anechoic demixing bears a close relationship to problems such as blind source separation and independent component analysis (cf. e.g., O'grady et al., 2005; Choi et al., 2005; Comon and Jutten, 2010). Numerous algorithms have been proposed to solve such problems for the most general case where the source functions $f_u(t)$ are assumed to be elements of relatively general function spaces. Specifically, for the under-determined case (in which the number of signals/sensors is smaller than the number of sources) well-known algorithms include information maximization approaches (Torkkola, 1996), time (Emile and Comon, 1998), frequency (Be'Ery and Yeredor, 2008), and time-frequency methods (Yilmaz and Rickard, 2004; Arberet et al., 2006; Cho and Kuo, 2009). The over-determined case is much more interesting for dimensionality reduction applications, but has been addressed less frequently. A notable exception includes Harshman et al. (2003), who developed an alternating least squares (ALS) algorithm for this problem (Shifted Factor Analysis). Their method was later revised and improved by Mørup and colleagues Mørup et al. (2007b) by exploiting the Fourier shift theorem and information maximization in the complex domain (SICA, Shifted Independent Component Analysis). More recently, Omlor and Giese (2011) developed a framework for blind source separation, starting from stochastic time-frequency analysis that exploited the marginal properties of the Wigner-Ville spectrum. Importantly, even though such algorithms are able to find general solutions to the demixing problem, they tend to be computationally expensive. Our framework restricts the search space by assuming that the source functions are band limited. This is equivalent to imposing a smoothness prior on the sources and speeds up the estimation process.

C DETAILS ABOUT FRAMEWORK VALIDATION

C.1 Generation of the simulated data

For the quantitative assessment of the algorithm's performance, we simulated kinematic and EMG data sets that were compatible with equations (1), (2), (3), (4) and (5). Each of these data sets approximated coarsely the properties of real biological signals. Each data set consisted of M-dimensional trajectories with T time steps and L repeated trials. Synthesized EMG signals were constrained to be non-negative, like real EMG signals after rectification and filtering. All generative models were based on a set of statistically independent temporal waveforms. These waveforms (source functions, or synergies) corresponded to the time-dependent combination coefficients $c_p^l(t)$ in model (1), to the temporal signals $s_p(t)$ in models (2) and (3) and (5), and to the components of the vector function in model in (4). For the generation of the unconstrained sources, we drew 100 random samples from a normal distribution (Matlab function "randn.m") and low-pass filtered with a Butterworth filter with normalized cut-off frequency equal to 0.15 (MATLAB functions "butter" and "filtfilt"). This procedure allowed to generate band-limited, smooth sources mimicking the typical properties of real kinematic or kinetic trajectories with a length of T = 100 time samples.

For the generation of EMG-like sources, we produced spike trains from a multi-dimensional stochastic renewal process (Kass and Ventura, 2001), and convolved them with a Gaussian function. The renewal process was a homogeneous Poisson process characterized by random inter-spike intervals drawn from an exponential distribution with mean $1/\lambda$, where the rate parameter of the Poisson process was given by λ = 40 Hz. Based on the random inter-spike intervals, spike trains with length T = 100 were generated. Each spike train was then convolved with a Gaussian filter kernel with a standard deviation of 8 discrete

time steps. The generated source signals were used to construct the synergies in the generative models (2), (3), (4) and (5). The weight vectors $\mathbf{w}$ in (1) and (5) were obtained by drawing M random samples from a uniform distribution over the interval [-40 40] for the unconstrained case, and from an exponential distribution with mean 20 for the cases with non-negativity constraints. Examples of generated primitives are shown in Fig 1. For kinematic (unconstrained) data sets based on model (2) and (3) the values of the coefficients c_{mp} were drawn from a uniform distribution over the interval [-20, 20]. For EMG-like data sets based on the models (2), (3), (4) and (5) the scaling coefficients were drawn from exponential distributions with mean 10. For all the models with time delays $\tau \neq 0$, the delays were drawn from exponential distributions with mean 20 and rounded to the nearest integer. The time delays sampled from this distribution with values larger than T = 100 were taken modulo to map them back to the interval [0, T-1]. Noisy data was derived by adding signal-dependent noise (Sutton and Sykes, 1967; Schmidt et al., 1979; Harris and Wolpert, 1998; van Beers et al., 2004) to the generated data. The noise was drawn from a Gaussian distribution with mean 0 and standard deviation $\sigma = \alpha\,|x(t)|$, where α is a scalar and $x(t)$ is the value of the noiseless data at the time instant t. The slope α was computed though an iterative procedure. Starting from $\alpha = 0$, its value was iteratively increased of a predefined increment until the level of the difference $1 - R^2$ (where the parameter R^2 describes the level of similarity between two data sets, see below) reached a predefined value. For each noiseless data set, three data sets were generated with $1 - R^2$ levels equal to 0.05, 0.15, 0.25 and 0.35). For each generative model, 20 noiseless data sets were simulated that were consistent with equations (1) to (5), randomly selecting synergies, scaling coefficients and time delays. The number of synergies P was always set to 4 and the number of simulated DOFs was 10. The number of simulated trials L was 25. The time duration of each trial was assumed to be $T_s = 1$ and the sampling frequency was set to 100 Hz.

C.2 Experimental kinematic and EMG data

We assessed the identification performance of each algorithm also on actual experimental kinematic and EMG data. The kinematic data set consisted of flexion angle trajectories of the body joints recorded from human actors walking with different emotional styles (neutral, happy and sad). These data were used in previous work on emotional gaits (Omlor and Giese, 2007; Roether et al., 2009; Endres et al., 2013). From this data set, unconstrained temporal primitives were identified with the FADA and the anechoic demixing algorithm. EMG data consisted of previously published recordings (d'Avella et al., 2006) obtained from 16 arm muscles during arm reaching movements. These muscle activation patterns were used to investigate the production of behaviors through combination of muscle synergies. The recorded EMG raw signals were digitally full-wave rectified, low-pass filtered (20 Hz cut-off) and integrated within time bins of 10 ms. All EMGs signals in the data set were resampled to fit a 75-point time window (0.75s).

C.3 Implementation details

To minimize the risk of finding local minima, we always ran the FADA algorithm 10 times on the same data set with different random initial conditions and we considered only the solutions that provided the lowest error in the reconstruction of the original data. To test whether these solutions actually represented points close to the global minimum, we computed the average similarity between the sets of primitives identified at the end of each run of the algorithm (see below for the definition of similarity). Indeed, a high level of similarity between these solutions can be considered as a strong sign that, with very high probability, these solutions are close to the optimal one. In the case, for instance, of an artificial mixture of non-negative temporal components based on model (2), we found that the average similarity between the identified primitives was very high (0.98 on a scale where 1 indicates perfect matching (see equation 16). This high level of similarity allows to rule out the hypothesis that the solutions provided by FADA represent local minima. For the identification of temporal, spatiotemporal or space-by-time primitives, the number of

harmonics K was always set according to the following procedure: We computed the average spectrum from all signals within the data and defined K as the closest integer that approximates the product of the signal duration T_s and the average band-width B of the data set. This number was always smaller thanthe limit K_{max} imposed by the the Nyquist-Shannon theorem. Differently, in the case of spatial primitives we always set $K = K_{max}$.

C.4 Benchmark identification algorithms

FADA-T was benchmarked against commonly used unsupervised learning methods for the extraction of synergies. For data based on the synchronous unconstrained generative models (1) and (2), we used the fastICA algorithm (Hyvärinen and Oja 1997; Hyvärinen 1999, function 'fastica.m' of the corresponding toolbox). We examined the performance of fastICA after reducing the dimensionality of the data using principal component analysis. For the fastICA algorithm, we found the level of similarity between original and identified synergies depended on the number of principal components and it reached the highest value when the number of principal components was equal to the number of synergies in the data. Based on this observation, we always set the number of principal components to the number of identified synergies. Non-negative matrix factorization (NMF — Lee and Seung, 1999, 2000) was used to identify the model parameters for synchronous mixture with non-negative components and mixing weights. In this case, we used the MATLAB function "nnmf.m", which implements the matrix multiplication update rule version of the algorithm introduced by Lee and Seung. For data relying on model (3), we used the anechoic demixing algorithm (AnDem — Omlor and Giese, 2011), and the shifted ICA algorithm (SICA — Mørup et al., 2007b). For anechoic demixing with non-negativity constraints, we used an anechoic NMF algorithm (AnNMF — Omlor and Giese, 2011) and the shifted NMF (sNMF — Mørup et al., 2007a). To extract time-varying synergies, we used the Spatiotemporal NMF (stNMF — d'Avella et al., 2003, 2006). Finally, we compared the performance of the FADA algorithm for the identification of temporal and spatial primitives from the space-by-time model with the performance of the Sample-based Non-negative Matrix tri-Factorization algorithm (sNM3F — Delis et al., 2014).

C.5 Assessment of algorithm performance

To quantify the goodness of fit of the models fitted by the considered identification algorithms, we used the coefficient of determination R^2, which measures the fraction of explained data variance:

$$R^2 = 1 - \frac{\sum_{l=1}^{L} \left\| \mathbf{X}^l - \mathbf{X}^l_{rec} \right\|_F^2}{\sum_{l=1}^{L} \left\| \mathbf{X}^l - \overline{\mathbf{X}^l} \right\|_F^2} \tag{17}$$

In the above equation, $\mathbf{X}^l$ represents the data matrix relative to trial l, $\mathbf{X}^l_{rec}$ the data matrix reconstructed by the fitted model, and $\overline{\mathbf{X}_l}$ the matrix of the time averages.

To assess the identification performance, defined as the ability of each algorithm to retrieve the ground-truth primitives, activation coefficients, and delays, we adopted a two-step procedure. We first matched ground-truth and estimated primitives following a greedy strategy based on the pairwise shape similarity; we then computed the similarities between the parameters (i.e., activation coefficients and delays) of the matched primitives. Specifically, to quantify the similarity S between the shapes of ground-truth $p(t)$ and estimated $\widehat{p}(t)$ primitives, we computed the maximum scalar product, over time delays τ, between $p(t)$ and $\widehat{p}(t - \tau)$:

$$S = \max_{\tau} \sum_{t} p(t) \cdot \widehat{p}(t - \tau) \tag{18}$$

Note that: (1) prior to computing S, we normalized the primitives to have unitary norm; (2) the similarity measure S takes on values between -1 and 1, where the value 1 indicates identical primitives shape; (3) importantly, for models without time delays, τ=0 and S becomes equivalent to the cosine similarity. Finally, to assess the similarity between ground-truth and identified activation coefficients and time delays, we computed the cosine similarity between the parameters of the matched primitives.

To facilitate the comparison of the similarity measures across different models and quantities (i.e., primitive shapes, activation coefficients, and delays), we further normalized such measures using an estimate of the average similarity between random pairs of model-specific realizations. Specifically, for each model and similarity measure, we first estimated the baseline similarity S_B by computing the average pairwise similarity in a set of 20 independent random samples of synergies, activation coefficients, and delays (where appropriate). We then computed the normalized similarity measures S_N as follows:

$$S_N = \frac{S - S_B}{1 - S_B} \tag{19}$$

This ensures that S_N approaches zero when the similarity between ground-truth and estimated quantities is comparable to the average similarity between random pairs of realizations; conversely, S_N approaches one as the estimates approach the ground truth.
